# Genetic variability and genome-wide association analysis of flavor and texture in cooked beans (*Phaseolus vulgaris* L.)

**DOI:** 10.1007/s00122-020-03745-3

**Published:** 2021-01-03

**Authors:** Amber Bassett, Kelvin Kamfwa, Daniel Ambachew, Karen Cichy

**Affiliations:** 1 Department of Plant, Soil and Microbial Sciences, Michigan State University, East Lansing, MI USA; 2 Department of Plant Science, University of Zambia, Lusaka, Zambia; 3 Southern Agricultural Research Institute, Hawassa, Ethiopia; 4 Department of Agricultural and Environmental Sciences, Tennessee State University, Nashville, TN USA; 5 Sugarbeet and Bean Research Unit, USDA-ARS, East Lansing, MI USA

## Abstract

**Key message:**

Cooked bean flavor and texture vary within and across 20 Andean seed types; SNPs are significantly associated with total flavor, beany, earthy, starchy, bitter, seed-coat perception, and cotyledon texture.

**Abstract:**

Common dry beans are a nutritious food recognized as a staple globally, but their consumption is low in the USA. Improving bean flavor and texture through breeding has the potential to improve consumer acceptance and suitability for new end-use products. Little is known about genetic variability and inheritance of bean sensory characteristics. A total of 430 genotypes of the Andean Diversity Panel representing twenty seed types were grown in three locations, and cooked seeds were evaluated by a trained sensory panel for flavor and texture attribute intensities, including total flavor, beany, vegetative, earthy, starchy, sweet, bitter, seed-coat perception, and cotyledon texture. Extensive variation in sensory attributes was found across and within seed types. A set of genotypes was identified that exhibit extreme attribute intensities generally stable across all three environments. seed-coat perception and total flavor intensity had the highest broad-sense heritability (0.39 and 0.38, respectively), while earthy and vegetative intensities exhibited the lowest (0.14 and 0.15, respectively). Starchy and sweet flavors were positively correlated and highest in white bean genotypes according to principal component analysis. SNPs associated with total flavor intensity (six SNPs across three chromosomes), beany (five SNPs across four chromosomes), earthy (three SNPs across two chromosomes), starchy (one SNP), bitter (one SNP), seed-coat perception (three SNPs across two chromosomes), and cotyledon texture (two SNPs across two chromosomes) were detected. These findings lay a foundation for incorporating flavor and texture in breeding programs for the development of new varieties that entice growers, consumers, and product developers alike.

**Electronic supplementary material:**

The online version of this article (10.1007/s00122-020-03745-3) contains supplementary material, which is available to authorized users.

## Introduction

Dry beans (*Phaseolus vulgaris* L.) are a nutritious food that serve as a staple in many majority-world countries (Akibode and Maredia 2011). Despite their global pervasiveness, they have limited consumption in the USA, with only 2.2 kg per capita consumed in 2019 (Parr and Lucier 2020). In the USA, primary breeding goals for dry beans include yield, processing quality, disease resistance, architecture, agronomic adaptation, stress tolerance, and grower friendliness, which encompasses traits that reduce labor and inputs required by growers (Kelly and Cichy 2012). Quality characteristics such as flavor and texture, however, have largely been overlooked in breeding programs. Quality is most commonly addressed through processing and the addition of sauces and flavors, especially to canned beans and bean products, often at the expense of nutritional value (Borchgrevink 2013; Roland et al. 2017; Gilham et al. 2018). Taste is a primary factor driving consumer purchasing decisions of food, which motivates food companies to invest heavily in this aspect of product development (William Blair 2016; IFIC 2019). Consumers are also very interested in clean labels and food products with few additives (Asioli et al. 2017).

Flavor and texture are characteristics that consumers consider when purchasing dry beans, influencing their decisions regarding market class and product type (Castellanos et al. 1997; Scott and Maideni 1998; Leterme and Carmenza Muñoz 2002; Eihusen and Albrecht 2007; Winham et al. 2019). However, for many consumers, beans are not palatable, and the beany flavor they impart when used as ingredients is often perceived as undesirable (Nachay 2017; Dougkas et al. 2019). Therefore, improving dry bean flavor and texture through breeding has the potential to increase consumer acceptance and utilization of beans and inclusion of beans as ingredients in products while appealing to consumers’ interest in flavor without many additives.

Flavor and texture are not typically evaluated prior to variety release in the USA, and this lack of focus on sensory quality may be limiting consumption of dry beans below their potential. A breeding approach to address flavor and texture in beans has not been explored in part due to the complexity and cost associated with sensory evaluations. Protocols have been developed for the preparation and evaluation of cooked bean samples as well as the training and maintenance of sensory panels (Koehler et al. 1987; Sanz-Calvo and Atienza-del-Rey 1999; Romero del Castillo et al. 2008, 2012), but these protocols are designed for few samples with plentiful seed and are not feasible to implement in breeding programs. The application of these sensory methods has identified genetic variability for texture and flavor acceptability (Koehler et al. 1987) and attribute intensities, including seed-coat perception, roughness, mealiness, and beany flavor (Rivera et al. 2013). This indicates that sensory quality can be addressed by harnessing the genetic variability present through breeding, provided appropriate phenotyping methods are available. There is a need for further evaluation of genetic variability for sensory attributes within *P. vulgaris* to understand the full range of attribute intensities available and to assess the genetic control of these attributes. These are important steps to develop a breeding program that incorporates flavor and texture.

For this study, a modified quantitative descriptive analysis approach was developed and applied to the screening of 1,960 samples for cooked bean flavor and texture. This approach was used to address three objectives: (1) to evaluate nine sensory attributes in 430 genotypes of a dry bean diversity panel grown in three locations, (2) to examine the relationships among sensory attributes, seed types, and cooking time, and (3) to identify genetic markers associated with sensory attributes across multiple locations.

## Materials and methods

### Germplasm

Subsets of the Andean Diversity Panel were grown and evaluated across three locations for this study. The genetic composition and germplasm origin of the ADP are described by Cichy et al. (2015) and included in Table S1. Only Andean genotypes were included in statistical and GWAS analyses. The Southern Agricultural Research Institute provided seeds from 373 Andean genotypes grown in Hawassa, Ethiopia, in Fall 2015, and the University of Zambia provided seeds from 251 Andean genotypes grown in Kabwe, Zambia, and 356 Andean genotypes grown in Lusaka, Zambia, in Spring 2018. Combined, a total of 430 genotypes were represented covering 20 seed types. Raw seed weights were recorded for each field rep as grams per 100 seeds.

In Hawassa, the ADP was grown during the main cropping season (July to October) in 2015 at the Hawassa Research Station, which has soil classified as Eutric Fluvisol with a pH of 7.0. The ADP genotypes were planted using an augmented design with genotypes arranged in 21 blocks, which each contained 13 test entries and 5 standard checks randomly allocated. Each genotype was planted in two-row plots with 0.4 m and 0.1 m inter-row and intra-row spacing, respectively. Each block was spaced 1 m apart. Fertilizer in the forms of urea (46% N, 0% P_2_O_5_, 0% K_2_O) and DAP (8% N, 46% P_2_O_5_, 0% K_2_O) was applied at a rate of 100 kg/ha.

In Kabwe, the ADP was grown at the Zambia Agricultural Research Institute Farm, which has soil classified as Ultisol and had a pH of 5.0. In Lusaka, the ADP was grown in the field during the rainy season in 2017 at the University of Zambia Research Farm, which has soil classified as fine loamy Isohyperthermic Paleustalf with a pH of 5.5. During the 2017 rainy season, a total of 850 mm of rain was received at the experimental site at the University Farm. In both Zambia locations, the ADP genotypes were planted using a randomized complete block design with two replications. In each replication, each genotype was planted in a single-row plot that was 4 M long with 0.60 M inter-row spacing. A compound fertilizer (10N:20P:10K) was applied to the experimental site at a rate of 100 kg Ha^−1^ just before planting.

Genotypes exhibiting extreme attribute intensities along with Red Hawk (dark red kidney) and Etna (cranberry) were grown at the Montcalm Research Center in MI in 2018. The soil type is Eutric Glossoboralfs (coarse-loamy, mixed) and Alfic Fragiorthods (coarse-loamy, mixed, frigid). Two-row plots 4.75 m long with 0.5 m spacing between rows were arranged in a randomized complete block design with two replications per genotype. Standard agronomic practices were followed as described in the MSU SVREC 2018 Research Report (Kelly et al. 2018).

### Cooking time evaluation

For each location, two replicates of 30 seed per genotype were equilibrated to 10–14% moisture in a 4 °C humidity chamber prior to evaluating for cooking time. For the seed from both locations in Zambia, each replicate corresponded to a field replicate. For the seed from Hawassa, Ethiopia, the single field replicate for each genotype was split to create two replicates. Each 30 seed sample was soaked for 12 h in distilled water and weighed prior to cooking time evaluation, performed using automated Mattson cookers (Wang and Daun 2005). Genotypes were cooked in a randomized order. Mattson cookers were loaded with soaked seeds and placed in boiling distilled water to cook. The Mattson cookers (Michigan State University Machine Shop, East Lansing, MI) use twenty-five 65 g stainless steel rods with 2-mm-diameter pins to pierce beans as they finish cooking in each well. As the pins drop, a custom software reports the cooking time associated with each pin. The cooking times were recorded, with the 80% cooking time regarded as the time required to fully cook each sample. Cooked samples were weighed, and total water uptake following cooking was calculated.

### Sensory evaluation

The ADP subsets from each location were evaluated in duplicate by four panelists each using a quantitative descriptive analysis (QDA) approach (Stone et al. 1974), in which each panelist independently evaluated samples using a non-consensus approach to limit group bias. QDA has been found to yield reproducible measurements with small differences for boiled dry beans, although it is typically applied to small numbers of samples due to the substantial time and personnel commitment it requires (McTigue et al. 1989). For the purposes of this study, the QDA approach was modified to make it feasible to screen hundreds of samples with replication using a small number of panelists, which is necessary for implementation in public breeding programs with limited resources. For each location, seeds were prepared for sensory evaluation in the same order as for cooking time evaluation. Four panelists were present at each sensory evaluation session, scheduled according to their availability. Sensory evaluation sessions were held daily until each genotype had been evaluated twice for each location. For the Ethiopia location, twenty genotypes were evaluated at each session. For the Zambia locations, twelve genotypes including cranberry (Etna) and dark red kidney (Red Hawk) bean controls grown at the Montcalm Research Center were evaluated at each session. Each sample was evaluated using 5-point attribute intensity scales (low → high intensity) for total, beany, vegetative, earthy, starchy, bitter, and sweet flavor intensities as well as seed-coat perception and cotyledon texture (Table S2). The scale for seed-coat perception ranged from imperceptible (1) to tough and lingering (5). For cotyledon texture, the scale ranged from mushy (1) to very gritty/firm (5). This sensory evaluation protocol was approved by the Institutional Review Board of Michigan State University (IRB#×16-763e Category: Exempt 6).

### Panel training and assessment

Panelists were recruited from the USDA-ARS (East Lansing, MI) and Michigan State University Dry Bean Breeding programs due to their familiarity with dry beans and their availability for long-term sensory evaluation projects.

**Fig. 1:**
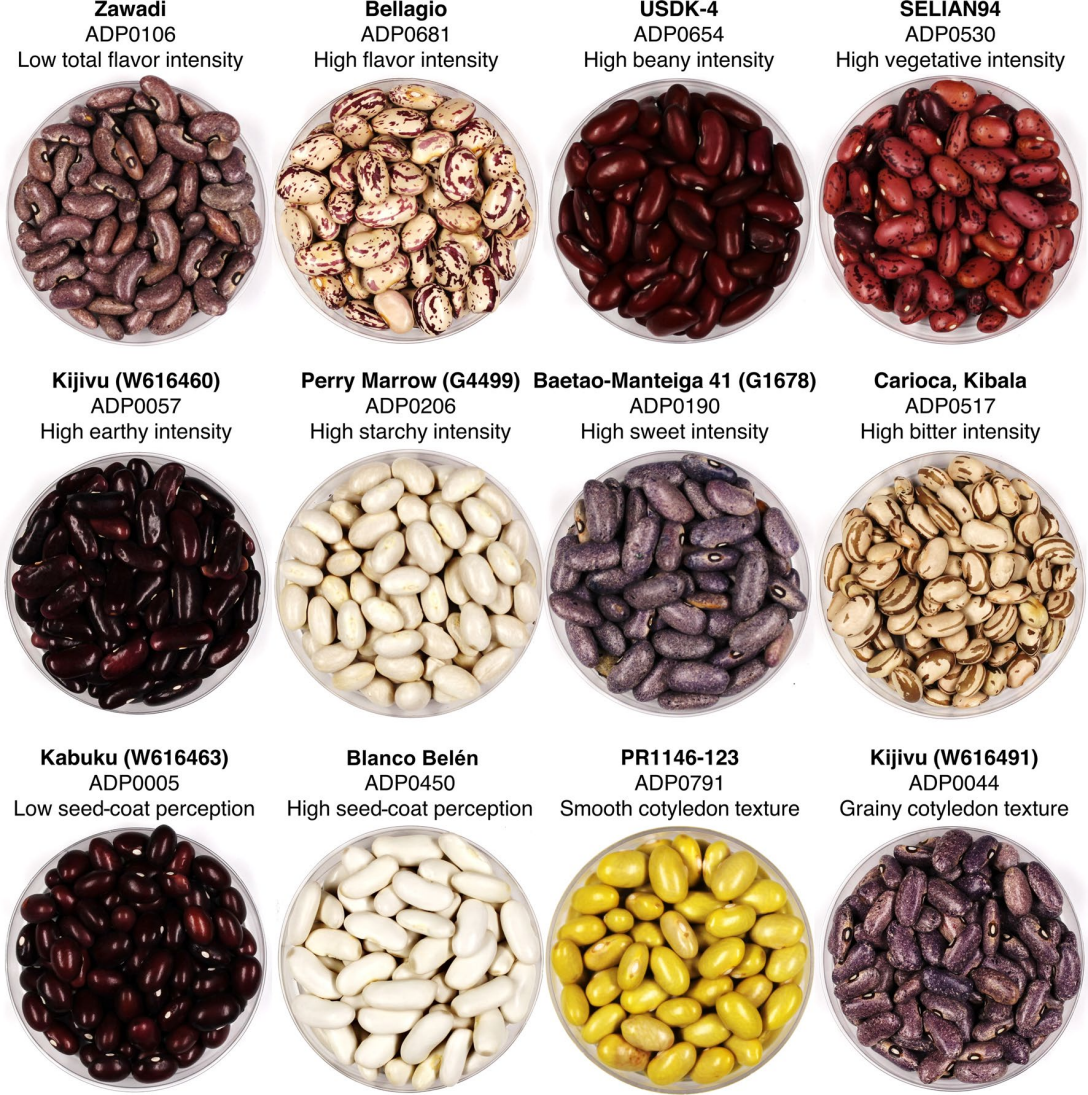
Images of the genotypes exhibiting extreme sensory attribute intensities identified from screening accessions of the Andean Diversity Panel grown in Hawassa, Ethiopia

An initial training session was conducted with eight panelists using a consensus approach to determine which attributes to evaluate and how to evaluate them. A diverse set of dry bean genotypes was selected from the USDA and MSU dry bean programs with the intention of exposing panelists to a wide range of attribute intensities. This initial set included black, cranberry, dark red kidney, great northern, Jacob’s cattle, navy, pink, pinto, small red, and yellow beans. Following screening of the ADP grown in Hawassa, Ethiopia, a training set of genotypes exhibiting extreme attribute intensities was developed (Table 1, Fig. 1). This set was used to train eleven panelists to rate the selected attributes prior to evaluating the ADP grown in the Zambia locations. For the sensory evaluation of the ADP from both Zambia locations, Red Hawk and Etna were used as controls. Red Hawk (Kelly et al. 1998), a dark red kidney bean, is a variety released by the Michigan State University dry bean breeding program. Etna (PI 546490), a cranberry bean, is a private variety developed by Seminis of Monsanto Vegetable Seeds.

**Table 1:** Genotypes exhibiting extreme sensory attribute intensities identified from screening accessions of the Andean Diversity Panel grown in Hawassa, Ethiopia

Genotype	ADP ID	Seed type	Region of origin	Sensory attribute
Zawadi	ADP0106	Purple speckled	Tanzania	Low total flavor intensity
Bellagio^a^	ADP0681	Cranberry	USA	High total flavor intensity
USDK-4^b^	ADP0654	Dark red kidney	USA	High beany intensity
SELIAN94	ADP0530	Red speckled	Tanzania	High vegetative intensity
Kijivu (W616460)	ADP0057	Dark red kidney	Tanzania	High earthy intensity
Perry Marrow (G4499)	ADP0206	White	USA	High starchy intensity
Baetao-Manteiga 41 (G1678)	ADP0190	Purple speckled	Brazil	High sweet intensity
Carioca, Kibala	ADP0517	Carioca	Angola	High bitter intensity
Kabuku (W616463)	ADP0005	Small red	Tanzania	Low seed-coat perception
Blanco Belén^c^	ADP0450	White	Ecuador	High seed-coat perception
PR1146-123^d^	ADP0791	Yellow	Puerto Rico	Smooth cotyledon texture
Kijivu (W616491)	ADP0044	Purple speckled	Tanzania	Grainy cotyledon texture

^a^Kelly et al. (2010)

^b^Miklas et al. (2004)

^c^Minchala et al. (2003)

^d^Beaver et al. (2016)

Panelists were trained over multiple sessions, first using a consensus approach to familiarize the panelists with the selected scales and sensory attributes. Panelists then practiced evaluating samples using a non-consensus approach to improve their familiarity with the scales and their sensory evaluation skills. Panelist performance was assessed via ANOVA with F_Genotype_ (*p* value < 0.05) indicating ability to discriminate and F_rep_ (*p* value > 0.05) indicating consistency (Meilgaard et al. 1999; Armelim et al. 2006). Sensory evaluation of each location commenced after successful training of each panelist. Following screening of the ADP from each location, panel performance was assessed as during training.

### Sample preparation for sensory evaluation

A standardized method for preparing boiled dry beans for sensory evaluation was previously developed (Romero del Castillo et al. 2012), but could not be applied in this study due to limited seed per genotype. Instead, the preparation method used by Mkanda et al. (2007) was modified to suit smaller seed volumes and a larger number of samples, as well as maintain consistent soaking time with the cooking evaluation method. In preparation for each sensory evaluation session, large tea bags filled with 12 h soaked seeds were boiled in distilled water for the cooking time determined by the Mattson cooker method, timed so they all finished cooking together. No salt was added. The cooked samples were poured into preheated (105 °C) ceramic ramekins, covered with aluminum foil, and placed in a chafing dish to maintain temperature. Samples were given a random letter code to mask their identity. Panelists were asked to refrain from wearing strong scents or eating during the hour before each session. Samples were served in randomized order out of the ceramic ramekins with a plastic spoon onto paper plates. Lemon water was made available as a palate cleanser (Han et al. 2010), and panelists were asked to drink water between samples.

### Statistics

PROC MIXED in SAS version 9.4 of the SAS System for Windows (SAS Institute Inc. Cary, NC, USA) was used to conduct ANOVAs for each recorded trait. For raw seed weight, soak water uptake, cooking time, and total water uptake traits, the fixed effects were genotype, location, and genotype by location with replicate as a random effect. For the sensory attribute intensity traits, the fixed effects were genotype, location, and genotype by location with rep, panelist nested in location, and session nested in location as random effects. Least squares estimates for sensory traits were calculated via the LSMeans statement in PROC MIXED for visualization of trait distributions with outliers excluded. To evaluate differences among seed types, ANOVAs were also performed with the seed type, location, and seed type by location as fixed effects and rep, panelist nested in location, and session nested in location as random effects.

To analyze all locations combined while minimizing environmental effects, best linear unbiased predictors (BLUPs) were generated for each trait using the lme4 package (Bates et al. 2015) in R (R Core Team 2017) with genotype, location, genotype by location, and rep nested in location as random effects. For sensory traits, panelist nested in location and session nested in location were also included as random effects. For analysis within individual locations, BLUPs were calculated for sensory traits with genotype, rep, panelist, and session included as random effects.

Broad-sense heritability (H2) was calculated on a family mean basis for each trait using the equation var(G)/(var(G) + (var (G * L)/no. loc) + (var(error)/no. loc * rep), where var is variance, G is genotype, and G * L is genotype by location, and no. loc is number of locations. Variance components were calculated using PROC VARCOMP in SAS version 9.4 with method = restricted maximum likelihood method (reml) (Holland et al. 2003). Pearson correlation coefficients among traits were determined with BLUPs from all locations combined using the cor function in R. Principal component analysis among traits was conducted via singular value decomposition of the centered and scaled BLUPs from all locations combined using the prcomp function in R.

### Genotyping

The ADP has been genotyped previously via genotyping by sequencing (GBS), and associated data including hapmaps are available at the Feed the Future—Development and Characterization of the Common Bean Diversity Panel (ADP) website (http://arsftfbean.uprm.edu/bean/) (Katuuramu et al. 2018). In brief, two GBS libraries were constructed at 364-plex and 137-plex as described by Elshire et al. (2011) with modifications described by Hart and Griffiths (2015). The raw sequencing data are available in association with BioProject accession number PRJNA290028 in the NCBI BioProject database (https://www.ncbi.nlm.nih.gov/bioproject/).

For this study, the raw sequence data were cleaned of adapters and trimmed for quality score ≥ 30 and minimum length ≥ 30 via Cutadapt (Martin 2011) and evaluated via FastQC (Andrews 2010). Cleaned reads were demultiplexed using the Next Generation Sequencing Eclipse Plugin (NGSEP) pipeline with NGSEP version 3.0.2 (Duitama et al. 2014; Perea et al. 2016), aligned to the Phaseolus vulgaris v2.1 genome (DOE-JGI and USDA-NIFA, http://phytozome.jgi.doe.gov/) using Bowtie 2 (Langmead and Salzberg 2012), and sorted using Picard (http://www.bioinformatics.babraham.ac.uk/projects/fastqc). Variant calling and annotation were performed via NGSEP. Raw SNPs were filtered to eliminate those with more than 90% missing data, and remaining missing data were imputed using FILLIN in Tassel 5.2.31 (Bradbury et al. 2007; Swarts et al. 2014).

### Genome-wide association

Genome-wide association analyses were performed with Bayesian-information and Linkage-disequilibrium Iteratively Nested Keyway (BLINK) (Huang et al. 2018) in R. BLINK has increased statistical power as compared to other methods and better controls for false negatives and false positives (Liu et al. 2016; Huang et al. 2018). Instead of using kinship, BLINK uses iterations to select a set of markers associated with a trait of interest, which are fitted as covariates. The first 3 principal components were determined using prcomp in R and included in each analysis to control for population structure. Single nucleotide polymorphisms (SNPs) with MAF < 0.05 or with more than two alleles were excluded from analysis. BLUPs were used in genome-wide association analyses for all locations combined and for sensory traits for individual locations, and means were used for analyses of all other traits for individual locations. BLINK does not report *R*^2^ for identified SNPs.

To support the BLINK findings, additional genome-wide association analyses were performed using a mixed linear model (MLM) approach in TASSEL v 5.2.31 (Bradbury et al. 2007). Kinship was calculated using normalized IBS (Yang et al. 2011), and the first 3 PCs were included to control for population structure. SNPs with MAF < 0.05 or with more than two alleles were excluded from analysis.

Manhattan plots and QQ plots were generated using the CMPlot R package (https://github.com/YinLiLin/R-CMplot), and significance levels were established using the false discovery rate (Benjamini and Hochberg 1995) for the BLINK analyses and using a Bonferroni correction based on the effective number of markers tested determined via SimpleM for the MLM analyses (Gao et al. 2008). When reporting significant SNPs from each GWAS analysis, the SNP with the lowest *p* value was chosen to represent each locus of interest.

## Results

### Sensory extremes

Twelve genotypes were identified which exhibited extreme sensory attributes (Table 1, Fig. 1). These genotypes were selected for training panelists because they exhibited the range of attributes likely present in the entire sample set. While the attribute intensities of these genotypes varied somewhat across the three locations, they collectively represented a large portion of the attribute intensity ranges that were observed, reflected by their least squares estimates across locations (Tables 2, S4). Significant genotype effects for each sensory attribute and insignificant rep effects indicated that the panelists were trained sufficiently to detect differences among genotypes and were consistent across reps despite significant panelist and session effects (Tables 2, S3).

**Table 2:** Least squares estimate (LSE), range, and coefficient of variation (CV) of sensory attribute intensities of the Andean Diversity Panel grown in three locations with ANOVA *p* values^a^ for genotype, location (Loc), and genotype by location and broad-sense heritability (H2) indicated

Trait	Location	LSE	Range	CV (%)	Genotype	Loc	Genotype × Loc	H2
Total flavor intensity								
	Hawassa, ET	2.8	1.6–3.7	14.4	< .0001	< .0001	< .0001	0.38
	Kabwe, ZM	3.4	2.2–4.4	12.6				
	Lusaka, ZM	3.4	2.0–4.5	13.3				
Beany intensity								
	Hawassa, ET	2.8	1.7–3.8	13.3	< .0001	NS	NS	0.30
	Kabwe, ZM	2.9	1.5–4.1	14.9				
	Lusaka, ZM	3.4	1.8–5.0	16.1				
Vegetative intensity								
	Hawassa, ET	2.0	1.1–3.4	17.8	< .0001	NS	0.0013	0.15
	Kabwe, ZM	2.4	1.3–3.7	16.0				
	Lusaka, ZM	2.6	1.6–4.0	16.4				
Earthy intensity								
	Hawassa, ET	2.0	1.2–3.0	15.7	< .0001	NS	NS	0.14
	Kabwe, ZM	2.1	1.2–3.2	17.0				
	Lusaka, ZM	2.1	1.2–3.4	18.6				
Starchy intensity								
	Hawassa, ET	3.2	2.2–4.4	10.4	< .0001	NS	NS	0.21
	Kabwe, ZM	3.2	2.1–4.0	11.7				
	Lusaka, ZM	3.2	2.2–4.1	12.2				
Sweet intensity								
	Hawassa, ET	1.7	1.0–3.5	21.2	< .0001	NS	< .0001	0.26
	Kabwe, ZM	1.9	0.9–3.2	21.2				
	Lusaka, ZM	1.8	0.8–3.1	21.2				
Bitter intensity								
	Hawassa, ET	1.6	0.8–3.5	22.0	< .0001	NS	NS	0.22
	Kabwe, ZM	1.5	0.8–3.0	22.0				
	Lusaka, ZM	1.4	0.5–2.8	24.6				
seed-coat perception								
	Hawassa, ET	3.0	1.6–4.4	13.3	< .0001	NS	< 0.0001	0.39
	Kabwe, ZM	3.1	2.2–4.1	13.1				
	Lusaka, ZM	3.0	1.6–4.1	13.8				
Cotyledon texture								
	Hawassa, ET	2.7	1.4–4.0	16.1	< .0001	0.0025	< .0001	0.31
	Kabwe, ZM	2.3	1.4–4.2	15.2				
	Lusaka, ZM	2.2	1.1–3.4	14.2				

^a^NS indicates non-significant *p* values at *α* = 0.05

### Sensory evaluation

The pin drop Mattson cooker was used to determine cooking times of the beans used in the sensory evaluation. The trained panel rated doneness of each cooked bean sample based on mouthfeel and concluded that the cooking times determined via the Mattson cooker equated to fully cooked samples (data not shown).

**Fig. 2:**
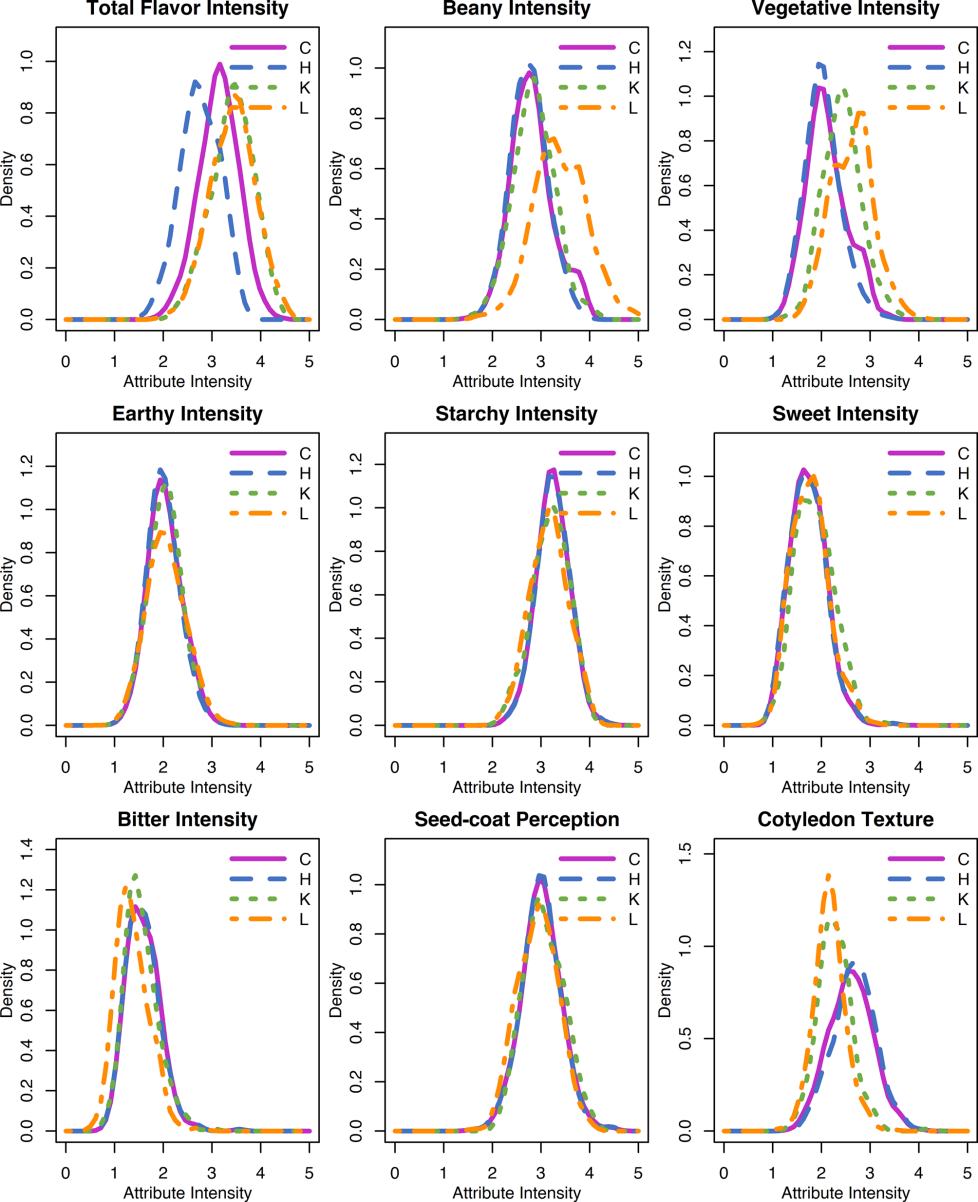
Density plots of least squares estimates of sensory attribute intensities for the Andean Diversity Panel for all locations combined (C); Hawassa, ET (H); Kabwe, Zambia (K); and Lusaka, Zambia (L)

Least squares estimates for sensory attribute intensities across all genotypes exhibited approximately normal distributions (Fig. 2). Genotype significantly affected all sensory attributes (*p* value < 0.05) (Table 2). Location significantly affected total flavor intensity and cotyledon texture (*p* value < 0.05), but was not a significant effect for other sensory attributes. Genotype by location significantly affected total flavor intensity, vegetative intensity, sweet intensity, seed-coat perception, and cotyledon texture (*p* value < 0.05).

Across all three locations, least squares estimates ranged 1.6–4.5 for total flavor intensity, 1.5–5.0 for beany intensity, 1.1–4.0 for vegetative intensity, 1.2–3.4 for earthy intensity, 2.1–4.4 for starchy intensity, 0.8–3.5 for sweet intensity, 0.5–3.5 for bitter intensity, 1.6–4.4 for seed-coat perception, and 1.1–4.2 for cotyledon texture. While panelists were able to differentiate among genotypes using 5-point scales, sensory attribute ranges did not exceed 3.2 in any single location, suggesting panelists did not make full use of the scales.

**Fig. 3:**
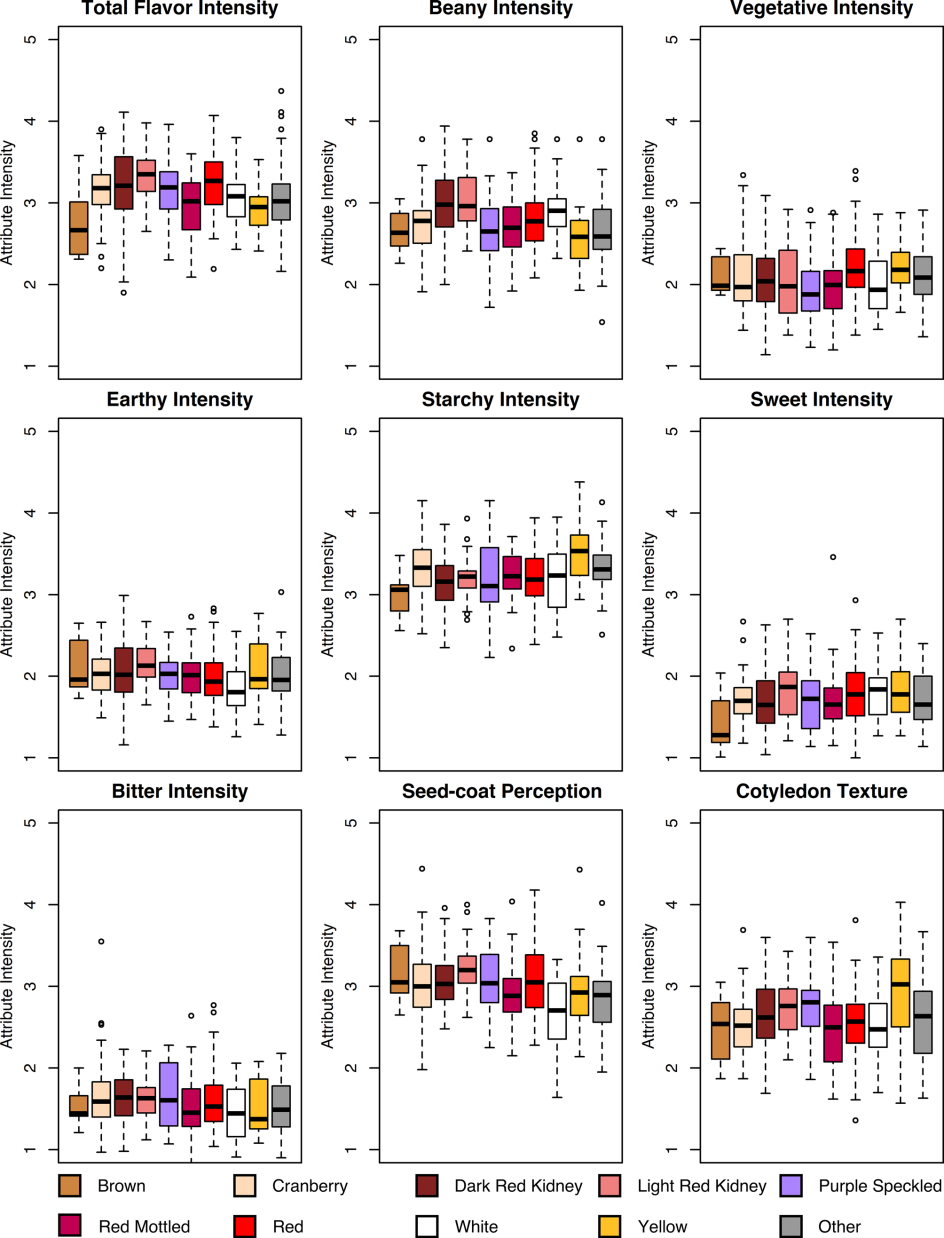
Boxplots of sensory attribute intensities separated by seed type. All boxplots are presented as least squares estimates averaged across all locations for seed types with *N* > 10, where “Other” includes the remaining seed types with *N* < 10

Twenty seed types were represented in the ADP, and seed type significantly affected all sensory attribute intensities (*p* value < 0.0001) (Table S5). However, large ranges of attribute intensities are observed for each seed type (Fig. 3), indicating flavor and texture vary within a seed type. Brown genotypes (*N* = 10) tended to vary the least across sensory attributes followed by light red kidney (*N* = 41), with cranberry (*N* = 63) and red mottled/red speckled (*N* = 80) varying the most. Earthy intensity followed by bitter intensity had the least variability across all seed types, and seed-coat perception and cotyledon texture had the most.

Broad-sense heritability for sensory attribute intensities was low, ranging from 0.14 to 0.39 (Table 2). seed-coat perception and total flavor intensity exhibited the highest broad-sense heritability (0.39 and 0.38), while earthy intensity and vegetative intensity exhibited the lowest (0.14 and 0.15).

### Cooking time evaluation

**Fig. 4:**
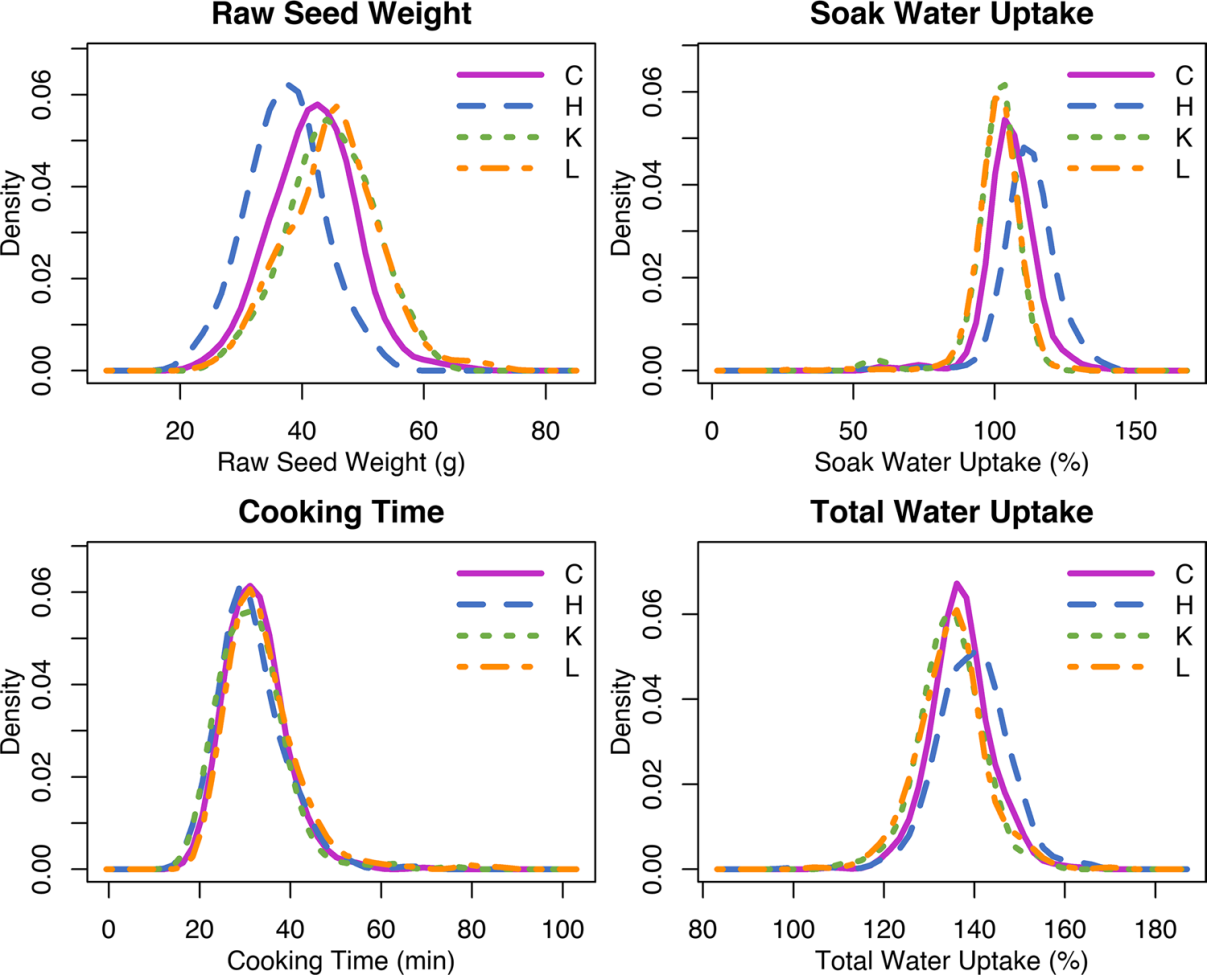
Density plots of means of raw seed weight, soak water uptake, cooking time, and total water uptake for the Andean Diversity Panel for all locations combined (C); Hawassa, ET (H); Kabwe, Zambia (K); and Lusaka, Zambia (L)

Genotype, location, and genotype by location significantly affected raw seed weight, soak water uptake, cooking time, and total water uptake (Table 3). The means and ranges of raw seed weight, soak water uptake, cooking time, and total water uptake varied across locations (Fig. 4). Across all 3 locations, raw seed weight ranged from 20.7–72.2 g per 100 seeds; soak water uptake ranged from 29.5–140.4%; cooking time ranged from 16.7–85.8 min; and total water uptake ranged from 100.4–169.7% (Table 3). Raw seed weight, soak water uptake, cooking time, and total water uptake exhibited approximately normal distributions (Fig. 4). Broad-sense heritability was moderate to high for raw seed weight (0.90), soak water uptake (0.85), cooking time (0.73), and total water uptake (0.65).

**Table 3:** Mean, range, and coefficient of variation (CV) of raw seed weight, soak water uptake, cooking time, and total water uptake of the Andean Diversity Panel grown in three locations with ANOVA *p* values for genotype, location (Loc), and genotype by location and broad-sense heritability (H2) indicated

Trait	Location	Mean	Range	CV (%)	Genotype	Loc	Genotype × Loc	H2
Raw seed weight (g per 100 seed)								
	Hawassa, ET	37.2	20.7–54.0	16.4	< .0001	< .0001	< .0001	0.90
	Kabwe, ZM	44.8	25.9–62.0	15.6				
	Lusaka, ZM	45.1	24.3–72.2	17.0				
Soak water uptake (%)								
	Hawassa, ET	112.1	51.9–140.4	8.9	< .0001	< .0001	< .0001	0.85
	Kabwe, ZM	100.3	54.0–118.6	9.3				
	Lusaka, ZM	101.0	29.5–128.1	8.7				
Cooking time (min)								
	Hawassa, ET	31.5	16.7–68.9	22.8	< .0001	< .0001	< .0001	0.73
	Kabwe, ZM	31.5	17.8–75.5	23.8				
	Lusaka, ZM	33.8	21.0–85.8	24.9				
Total water uptake (%)								
	Hawassa, ET	139.5	100.4–165.2	5.7	< .0001	< .0001	< .0001	0.65
	Kabwe, ZM	134.8	110.7–156.2	5.1				
	Lusaka, ZM	135.0	105.0–169.7	5.6

### Correlations and PCA

**Fig. 5:**
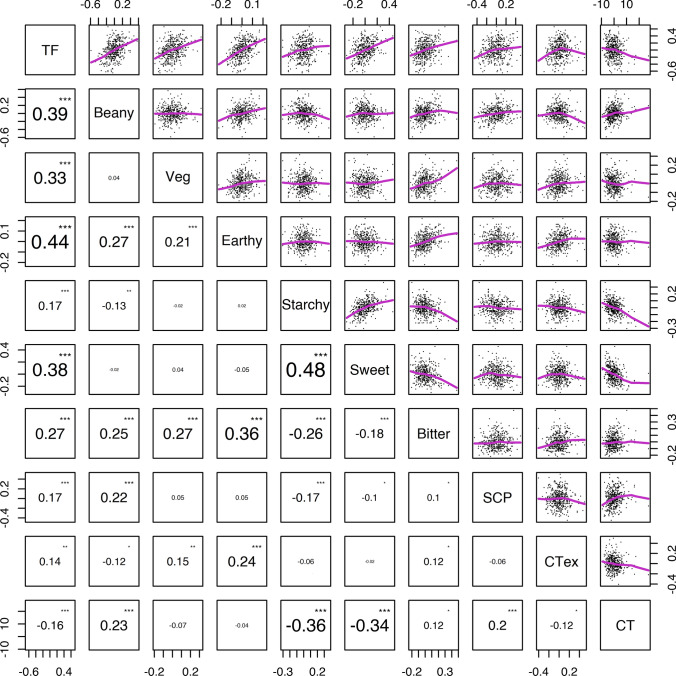
Pairwise comparison matrix of cooking time (CT), total flavor intensity (TF), beany intensity (beany), vegetative intensity (veg), earthy intensity (earthy), starchy intensity (starchy), sweet intensity (sweet), bitter intensity (bitter), seed-coat perception (SCP), and cotyledon texture (CTex). Pearson correlation coefficients were calculated using BLUPs and are indicated in the lower left, and scatterplots for each pairwise comparison with LOWESS regression lines are shown in the upper right. *p* values are indicated by asterisks, where *, **, and *** represent < 0.05, < 0.01, and < 0.001, respectively

Many significant correlations were observed among sensory attribute intensities and cooking time (Fig. 5). However, all of the significant correlations observed are weak to moderate, with the strongest correlation coefficient not exceeding an absolute value of 0.5. Weak correlations among traits suggest that sensory attributes and cooking time can be packaged together in multiple ways by breeders to develop varieties suited for consumer acceptance.

Total flavor intensity correlated positively with all other sensory attributes such that earthy (*R* = 0.44, *p* value < 0.0001), beany (*R* = 0.39, *p* value < 0.0001), sweet (*R* = 0.38, *p* value < 0.0001), vegetative (*R* = 0.33, *p* value < 0.0001), bitter (*R* = 0.27, *p* value < 0.0001), and starchy (*R* = 0.17, *p* value = 0.0004) intensity all increased with total flavor intensity. The correlations between total flavor intensity and seed-coat perception (*R* = 0.17, *p* value = 0.0003) and cotyledon texture (*R* = 0.14, *p* value = 0.0050) indicate that more flavor is associated with tougher, lingering seed-coats and grittier, firmer cotyledons in fully cooked seeds. Total flavor intensity was negatively correlated with cooking time (*R* = − 0.16, *p* value = 0.0009), suggesting that genotypes with shorter cooking times have more total flavor, potentially due to less time for leaching during the cooking process.

Individual sensory attributes also correlated with one another. Genotypes with high beany intensity tended to be somewhat earthy (*R* = 0.27, *p* value < 0.0001) and bitter (*R* = 0.25, *p* value < 0.0001) and less starchy (*R* = − 0.13, *p* value = 0.0073). Genotypes with high vegetative intensity also tended to be somewhat earthy (*R* = 0.21, *p* value < 0.0001) and bitter (*R* = 0.27, *p* value < 0.0001). Genotypes with high earthy intensity were bitter (*R* = 0.36, *p* value < 0.0001) as well as beany and vegetative. Genotypes with high starchy intensity were notably sweet (*R* = 0.48, *p* value < 0.0001), less bitter (*R* = − 0.26, *p* value < 0.0001), and less beany. Genotypes with high sweet intensity were also observed as being less bitter (*R* = − 0.18, *p* value = 0.0002). Genotypes with high bitter intensity were somewhat beany, vegetative, and earthy and less starchy or sweet. Genotypes with tougher seed-coats were beany (*R* = 0.22, *p* value < 0.0001) and bitter (*R* = 0.10, *p* value = 0.0386) and less starchy (*R* = − 0.17, *p* value = 0.0003) or sweet (*R* = − 0.10, *p* value = 0.0343). Genotypes with grittier/firmer cotyledon texture were vegetative (*R* = 0.15, *p* value = 0.0024), earthy (*R* = 0.24, *p* value < 0.0001), and bitter (*R* = 0.12, *p* value = 0.0147) and less beany (*R* = − 0.12, *p* value = 0.0167).

Cooking time also correlated with individual sensory attributes. Faster-cooking genotypes were starchy (*R* = − 0.36, *p* value < 0.0001) and sweet (*R* = − 0.34, *p* value < 0.0001) and had smoother cotyledon texture (*R* = − 0.12, *p* value = 0.0123), while slower cooking genotypes were beany (*R* = 0.23, *p* value < 0.0001) and bitter (*R* = 0.12, *p* value = 0.0167) and had tougher seed-coats (*R* = 0.2, *p* value < 0.0001).

**Fig. 6:**
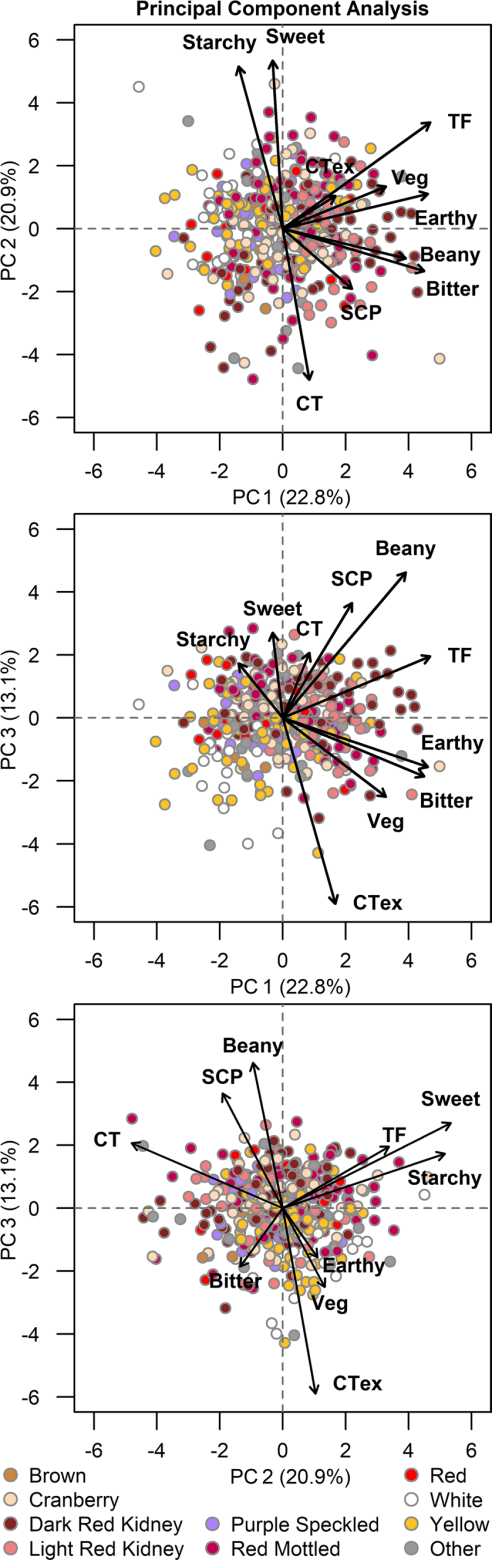
Principal component analysis biplots with each genotype colored by seed type and loadings indicated for total flavor intensity (TF), beany intensity (Beany), vegetative intensity (Veg), earthy intensity (Earthy), starchy intensity (Starchy), sweet intensity (Sweet), bitter intensity (Bitter), seed-coat perception (SCP), cotyledon texture (CTex), and cooking time (CT)

For the PCA, the first three principal components (PCs) explained about 60% of the variation (Fig. 6). The first PC separated the genotypes approximately by total flavor, earthy, and bitter intensities and represented almost a quarter of the variation (22.8%). The second PC represented a similar amount of the variation (20.9%) and separated the genotypes by starchy and sweet intensities and cooking time. The third PC represented about an eighth of the variation (13.1%) and separated the genotypes approximately by beany and vegetative intensities, cotyledon texture, and seed-coat perception. The remaining PCs accounted for 8.9, 8.4, 6.7, 6.2, 5.5, 4.4, and 3.1% of the variance, respectively (data not shown). The PCA biplots highlight the positive relationships between starchy and sweet intensities, vegetative and earthy intensities, and beany intensity and seed-coat perception and the negative relationship between cooking time and sweet and starchy intensities.

Each genotype within the PCA biplots is colored by seed type, which reveals substantial variation within seed type. All seed types are spaced somewhat evenly across the biplots except for the white seed type. White genotypes tend to cluster near starchy and sweet and away from cooking time and seed-coat perception, which indicates that white genotypes tend to be starchy and sweet with shorter cooking times. For the first two PCs, dark red kidney, light red kidney, and red mottled genotypes are distributed somewhat closer toward loadings for total flavor intensity, vegetative intensity, earthy intensity, and cotyledon texture, and purple speckled genotypes are distributed somewhat away, but the clustering is very loose.

### Genome-wide association mapping

Across the 430 Andean genotypes evaluated in this study, 31,273 SNPs remained after imputing and filtering. For each location, a similar number of SNPs were used in GWAS: 29,926 SNPs from Hawassa, Ethiopia (*N* = 373), 29,545 SNPs from Kabwe, Zambia (*N* = 251), and 31,484 SNPs from Lusaka, Zambia (*N* = 356).

**Fig. 7:**
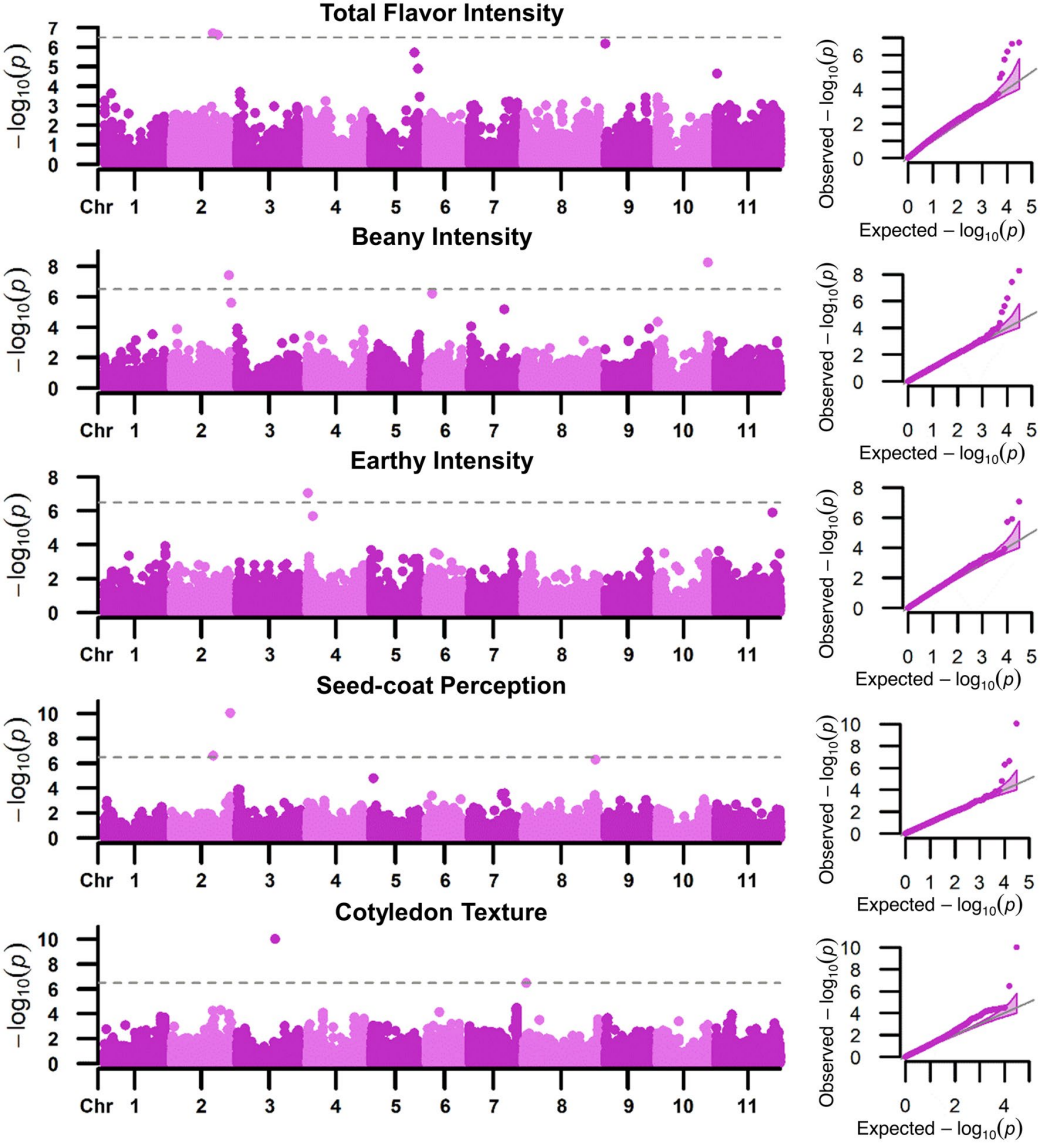
Manhattan and QQ plots for total flavor intensity, beany intensity, earthy intensity, seed-coat perception, and cotyledon texture of the Andean Diversity Panel with mapping conducted using BLINK with BLUPs from all locations combined. The gray dashed line is the *α* = 0.05 FDR

**Fig. 8:**
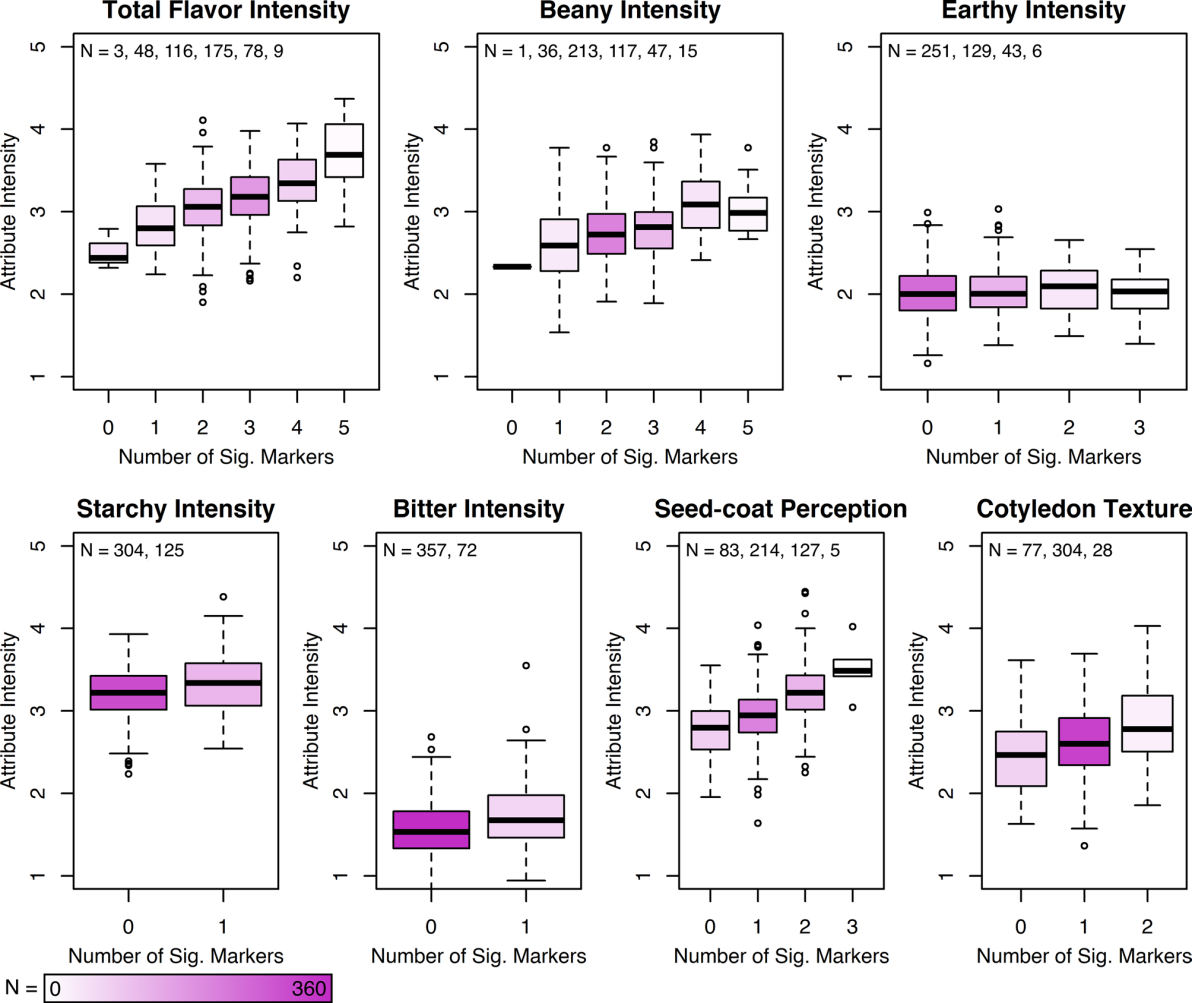
Phenotypic effects of carrying the indicated number of significant markers conferring a positive effect for each sensory attribute. Phenotypic values represent all locations combined as means of least squares estimates from Hawassa, Ethiopia; Kabwe, Zambia; and Lusaka, Zambia. N is the number of individuals in each boxplot

Across all locations combined, significant SNPs were identified using BLINK and MLM for several sensory attributes, including total flavor intensity, beany intensity, earthy intensity, starchy intensity, bitter intensity, seed-coat perception, and cotyledon texture (Fig. 7, S1). Significant SNPs detected for sensory traits were not consistent across the BLINK and MLM analyses methods, except for cotyledon texture (Table 4). MLM identified fewer significant SNPs overall, as expected due to its lower power and poor control of false negatives as compared to BLINK (Liu et al. 2016; Huang et al. 2018). For each sensory attribute with significant marker associations, an increase in the number of alleles conferring positive effects corresponded to an increase in mean attribute intensity (Fig. 8).

**Table 4:** GWAS significant markers associated with sensory attribute intensities with marker, chromosome (Chr), position, *p* values, minor allele frequency (MAF), major and minor alleles (Maj/Min), significance (Sig), and method indicated

Trait	Marker	Chr	Position^a^		*p* value		MAF		Maj/Min^b^	Sig^c^	Method
Total flavor intensity											
	S01_5952237	1	5,952,237		1.87E−05		0.06		**G**/T	*	MLM
	S02_34288083	2	34,288,083		1.94E−07		0.27		**A**/G	***	BLINK
	S02_38579748	2	38,579,748		2.31E−07		0.07		T/**A**	***	BLINK
	S05_36225444	5	36,225,444		1.91E−06		0.15		C/**T**	**	BLINK
	S05_39325999	5	39,325,999		1.23E−05		0.28		**C**/T	*	BLINK
	S09_235919	9	235,919		6.53E−07		0.10		C/**T**	***	BLINK
Beany intensity											
	S02_47727086	2	47,727,086		3.67E−08		0.22		G/**C**	***	BLINK
	S02_49605939	2	49,605,939		2.48E−06		0.06		**C**/T	**	BLINK
	S06_5174714	6	5,174,714		6.15E−07		0.14		G/**T**	***	BLINK
	S07_28996873	7	28,996,873		6.66E−06		0.37		**G**/T	**	BLINK
	S10_42475118	10	42,475,118		5.51E−09		0.15		**T**/C	***	BLINK
Earthy intensity											
	S04_528286	4	528,286		8.63E−08		0.07		C/**T**	***	BLINK
	S04_4661131	4	4,661,131		1.98E−06		0.19		G/**A**	**	BLINK
	S11_47172346	11	47,172,346		1.23E−06		0.30		A/**T**	**	BLINK
Starchy intensity											
	S01_42652564	1	42,652,564		5.42E−06		0.30		G/**A**	**	MLM
Bitter intensity											
	S01_51119029	1	51,119,029		1.47E−05		0.20		C/**T**	*	MLM
seed-coat perception											
	S02_34629777	2	34,629,777		2.43E−07		0.10		A/**C**	***	BLINK
	S02_48936819	2	48,936,819		9.06E−11		0.26		C/**T**	***	BLINK
	S08_60104671	8	60,104,671		4.90E−07		0.23		**C**/G	***	BLINK
Cotyledon texture											
	S03_31659572	3	31,659,572		9.43E−11		0.18		**G**/T	***	BLINK, MLM
	S08_2356200	8	2,356,200		3.32E−07		0.08		A/**G**	***	BLINK, MLM

^a^Position is based on the *P. vulgaris* v2.1 reference genome (DOE-JGI and USDA-NIFA, http://phytozome.jgi.doe.gov/)

^b^Alleles in bold confer a positive effect on the indicated trait

^c^Significance is indicated by asterisks, such that *, **, *** indicate significance at *α* = 0.1, *α* = 0.05, *α* = 0.01 using the false discovery rate for the BLINK method and a Bonferroni correction based on the effective number of markers determined using the SimpleM algorithm for the MLM method

For total flavor intensity, six significant SNPs were identified on Pv01, Pv02, Pv05, and Pv09 (Table 4). Genotypes with five significant SNPs conferring positive effects had a mean total flavor intensity rating 1.2 higher than those with no positive significant SNPs (Fig. 8). There were no genotypes with all six positive significant SNPs. For beany intensity, five significant SNPs were identified on Pv02, Pv06, Pv07, and Pv10 (Table 4). Genotypes with all five significant SNPs conferring positive effects had a mean beany intensity rating 0.8 higher than those with no positive significant SNPs (Fig. 8). For earthy intensity, three significant SNPs were identified on Pv04 and Pv11 (Table 4). Genotypes with all three significant SNPs conferring positive effects had a mean earthy intensity rating about equal to those with no positive significant SNPs when presented as means of least squares estimates (Fig. 8) and slightly increased (0.1) when presented as means of BLUPs (data not shown). Starchy intensity had one significant marker on Pv01 (S01_42652564) (Table 4). Genotypes with the significant marker conferring a positive effect had a mean starchy intensity rating 0.1 higher than those without the positive significant marker (Fig. 8). Bitter intensity also had one significant marker on Pv01 (S01_51119029) (Table 4). Genotypes with the significant marker conferring a positive effect had a mean bitter intensity rating 0.2 higher than those without the positive significant marker (Fig. 8). For seed-coat perception, three significant SNPs were detected on Pv02 and Pv08 (Table 4). Genotypes with all three significant SNPs conferring positive effects had a mean seed-coat perception rating 0.7 higher than those with no positive significant SNPs (Fig. 8). For cotyledon texture, two significant SNPs were detected on Pv03 and Pv08 (Table 4). Genotypes with both significant SNPs conferring positive effects had a mean cotyledon texture rating 0.4 higher than those with no positive significant SNPs (Fig. 8).

For each individual location, significant SNPs were also identified using BLINK for total flavor intensity, beany intensity, earthy intensity, and seed-coat perception (Table S6). MLM was not performed for individual locations. The identified SNPs somewhat reflect the findings for all locations combined, but largely point to different SNPs relevant for specific locations. For total flavor intensity, a total of fifteen significant SNPs were identified on Pv02, Pv03, Pv04, Pv05, and Pv11 in the samples from Hawassa Ethiopia; Pv03, Pv08, Pv09, Pv10, and Pv11 in the samples from Kabwe, Zambia; and Pv05, Pv06, and Pv10 in the samples from Lusaka, Zambia (Table S6, Fig. S2). For beany intensity, a total of six significant SNPs were identified on Pv10 and Pv11 in the samples from Kabwe, Zambia, and Pv02, Pv06, Pv10, and Pv11 in the samples from Lusaka, Zambia (Table S6, Fig. S3). For earthy intensity, a total of three significant SNPs were identified on Pv04 in the samples from Kabwe, Zambia, and Pv02 and Pv11 in the samples from Lusaka, Zambia (Table S6, Fig. S4). For seed-coat perception, a total of five significant SNPs were identified on Pv02 and Pv05 in the samples from Hawassa, Ethiopia; Pv05 in the samples from Kabwe, Zambia; and Pv02 and Pv07 in the samples from Lusaka, Zambia (Table S6, Fig. S5).

Across all locations combined, significant SNPs were identified using BLINK and MLM for raw seed weight, soak water uptake, cooking time, and total water uptake (Figs. S6, S7). Both methods identified different SNPs, with some overlap for raw seed weight and soak water uptake (Table S7). MLM identified fewer significant SNPs overall, as was the case for the sensory attributes.

For raw seed weight, fifteen significant SNPs were identified on Pv01, Pv02, Pv03, Pv04, Pv05, Pv06, Pv08, Pv09, and Pv11 (Table S7). Genotypes with thirteen significant SNPs conferring positive effects had a mean raw seed weight 31 g per 100 seeds higher than those with only three positive significant SNPs (Fig. S8). For soak water uptake, seventeen significant SNPs were identified on Pv02, Pv03, Pv04, Pv05, Pv07, Pv08, Pv10, and Pv11 (Table S7). Genotypes with fifteen significant SNPs conferring positive effects had a mean soak water uptake 64% higher than those with only four positive significant SNPs (Fig. S8). For cooking time, eleven significant SNPs were identified on Pv03, Pv04, Pv06, Pv07, Pv08, and Pv11 (Table S7). Genotypes with nine significant SNPs conferring negative effects had a mean cooking time 23 min faster than those with three or fewer negative significant SNPs (Fig. S8). For total water uptake, five significant SNPs were identified on Pv03, Pv04, Pv09, and Pv11 (Table S7). S04_30764016 was associated with both soak water uptake and total water uptake. Genotypes with all five significant SNPs conferring positive effects had a mean total water uptake 10% higher than those with one or fewer positive significant SNPs (Fig. S8).

## Discussion

The modified QDA approach used in this study successfully detected differences among genotypes for the purposes of identifying extremes, evaluating the relationships among sensory attributes and seed type, and performing genome-wide association analyses to reveal SNPs associated with sensory attributes. Although significant panelist effects were identified, these effects are not concerning because QDA does not rely on consensus among panelists. However, limited use of the scales by the panelists prevents detection of small differences between samples. This can be remedied by increasing the size of the scales or using 15 cm line scales that allow for continuous rather than discrete ratings. As for panelists, differences among sessions are expected and can be accounted for in the ANOVAs and by using BLUPs where appropriate. Genotypes exhibiting extreme attribute intensities were identified and successfully used for training panelists for sensory evaluation. These genotypes could serve as a training set for future sensory research or for training sensory panels for germplasm evaluation in breeding programs.

Production environment and crop management practices have previously been identified as factors affecting sensory quality (Mkanda et al. 2007; Ferreira et al. 2012), which complicates efforts to understand and breed for sensory quality in beans. The location and genotype by location effects were significant for many of the sensory attributes in this study, supporting these findings. Differences among locations were also apparent in density plots for some flavor and texture attributes. Despites small fluctuations in sensory profile across locations, the genotypes exhibiting extreme sensory attribute intensities remained extreme for their attribute of interest in each location. This suggests that differences across location affect magnitude of sensory attribute intensities, but do not substantially alter sensory attribute intensities relative to each other.

Many significant correlations were identified among flavor, texture, and cooking time, although correlation coefficients were generally weak, suggesting that traits can combine in multiple ways. Sweet and starchy intensity were the two most strongly correlated attributes, and the loadings for these attributes were positioned near each other in the PCA biplots, away from other attributes. White seeds were generally sweet and starchy, but otherwise, few trends were identified in regard to seed type, which indicates that seed type does not define the sensory profile of a genotype. This supports a previous study that found similarities in morphology and genetic background do not indicate similarity of sensory attributes among genotypes (Rivera et al. 2013). The genetic variability existing within seed type could be harnessed to achieve a target sensory profile and ensure greater consistency and uniformity of flavor and texture. In addition, fast cooking time could be targeted without substantially influencing sensory profile, which would address another major factor influencing consumer purchasing decisions (Leterme and Carmenza Muñoz 2002; Eihusen and Albrecht 2007; Winham et al. 2019).

Many SNPs significantly associated with flavor and texture were identified using BLINK and MLM, and they appear to confer minor effects, highlighting the complexity of the genetics underlying these traits. Significant SNPs varied for each individual location, emphasizing the importance of location in expression of genetic variability for sensory attributes. The significant SNPs identified have not been previously associated with sensory attributes as this is the first study of its kind in beans. No significant SNPs were associated with vegetative or sweet intensities, but alternative approaches such as QTL mapping or genomic prediction with a population of related individuals may provide increased power to detect relevant loci for these traits. Other studies in fruits have successfully used volatiles and instrumental measures in GWAS as proxies for flavor and texture, allowing for easier phenotyping and in some cases higher heritability than traits evaluated via descriptive panels (Zhang et al. 2015; Amyotte et al. 2017; Bauchet et al. 2017; Zhao et al. 2019). However, volatiles and instrumental measures do not always successfully predict flavor and texture as it is perceived by a descriptive panel (Amyotte et al. 2017), and for dry beans, little is known about how volatiles or other measures relate to flavor and texture. The screening of the ADP performed in this study provides a resource for future population development to further understanding of the genetic control of sensory attributes and how volatiles and instrumental measurements relate to sensory attributes.

One of the unique flavor characteristics found in dry beans and other legumes consumed as seeds is the “beany” flavor, which has proven a challenge to define and is often described as an “off” flavor in products using beans as ingredients (Kinsella 1979; Bott and Chambers 2006; Hooper et al. 2019). One study defined the flavor as undesirable, with multiple contributing volatiles (Vara-Ubol et al. 2004). In soybean, significant SNPs have been associated with volatiles contributing to beany flavor, and some of these SNPs are present in regions syntenic with dry bean chromosomes where SNPs associated with beany flavor were identified in this study (Schmutz et al. 2014; Xia et al. 2019a, b; Wang et al. 2020). In particular, the end of Pv02 where S02_47727086 and S02_49605939 are located is syntenic with soybean chromosomes 5 and 8 (Schmutz et al. 2014). Using Minimap2 (Li 2018) and the soybean reference genome (Williams 82) from SoyBase (Grant et al. 2009), the 50 kb regions around S02_47727086 and S02_49605939 align near rs39728576 and rs4039554, respectively, markers on soybean chromosome 5 and 8 associated with hexanal content in soybean (Wang et al. 2020).

Off-flavors in soy products are generated by lipoxygenases, primarily Lipoxygenase-2, or the oxidative rancidity of unsaturated fatty acids (Wolf et al. 1971; Kim et al. 2004). Markers linked to Lipoxygenase-2 are available and in use for breeding efforts targeting the reduction of beany flavor in soybean (Lenis et al. 2010; Talukdar and Shivakumar 2016). Several lipoxygenase genes are located within a megabase of S07_28996873 and S10_42475118 (http://phytozome.jgi.doe.gov/). In addition, a single lipoxygenase is located within three megabases of S06_5174714. While some lipoxygenases are present on Pv02, they are not close to S02_47727086 or S02_49605939.

It is not yet understood whether beany flavor in boiled beans translates to off-flavor in products made using beans as ingredients. In addition, consumer preference as it relates to sensory attribute intensities has not been explored for boiled beans beyond a general preference for beans that are sweet and soft when fully cooked (Mkanda et al. 2007). Further research relating consumer acceptability to attribute intensities in boiled beans as well as products using beans as ingredients could allow breeders to identify target sensory profiles for different seed types or varieties intended for use as ingredients.

In regard to raw seed weight, soak water uptake, cooking time, and total water uptake, many significant SNPs were identified in association with these traits as well via BLINK and MLM. Most of the SNPs identified were novel, but some were proximal to QTL and markers identified in previous studies. Of particular interest, S11_10805992, which was significantly associated with cooking time, is near a QTL identified for cooking time by Berry et al. (2020). S02_47837868, S03_50652595, S03_51140861, S04_30764016, S07_3919560, S10_37637761, which were significantly associated with soak water uptake, appear to be supported by hydration coefficient and water absorption QTL previously identified (Pérez-Vega et al. 2010; Cichy et al. 2014; Kelly and Bornowski 2018; Sandhu et al. 2018).

While broad-sense heritability for each sensory attribute was generally low, heritability could be improved in the context of a breeding program by screening only promising lines with greater replication. This could allow for better understanding of panelists and session effects and a balanced statistical design while maintaining a manageable time and personnel commitment. If fewer samples are evaluated each session, sensory fatigue could be reduced, allowing for better detection of small differences between samples. Potential alternative methods for screening sensory attributes could also be explored, including screening volatile profiles via GC–MS and collecting NIR spectra. NIR spectra of both raw seeds and cooked and dried seeds have been analyzed for their ability to predict beany flavor, mealiness, seed-coat roughness, and seed-coat brightness, although correlations between NIR spectra and these attributes were poor for raw beans (Plans et al. 2014). Using alternative methods for screening sensory attributes could increase the throughput of sensory profile characterization, but more research is needed to identify predictive measurements.

## Conclusion

This study lays a foundation for incorporating sensory quality traits into dry bean breeding programs. The broad range of sensory attribute intensities observed across and within seed types indicates a lack of uniformity within seed type, but also a wealth of genetic variability for sensory quality. This presents an opportunity for specific sensory profiles to be defined for each seed type. The limited correlations among sensory attributes indicate that they can combine in multiple ways, suggesting it is feasible to target specific sensory profiles according to consumer preference. Using the modified QDA approach to screen materials and the significant SNPs identified for flavor and texture attributes, breeders could continue to improve agronomic traits without sacrificing desirable sensory quality. The set of genotypes exhibiting extreme sensory attribute intensities identified in this study can be used for panel training as well as future work exploring sensory attributes and consumer preference. In addition, further understanding of sensory profiles suitable for bean products would allow varieties to be developed for use as ingredients, increasing the chance of success for bean products on the market. Improving flavor and texture in dry beans can ensure beans are appreciated as a delicious and tasteful component of a healthful diet in all the versatile ways consumers choose to eat them.

## Electronic supplementary material

Below is the link to the electronic supplementary material.

Supplementary file2 (PDF 1206 kb)

Supplementary file1 (XLSX 350 kb)

## Data Availability

The source information for the genotypes used in this study is included in Table S1. The phenotypic data collected in this study are provided in a supplementary file. The raw sequencing data used in this study are available in association with BioProject accession number PRJNA290028 in the NCBI BioProject database (https://www.ncbi.nlm.nih.gov/bioproject/).
