# Supplementary material for: Genetic variability and genome-wide association analysis of flavor and texture in cooked beans (Phaseolus vulgaris L.)
Source: Theor Appl Genet. 2021 Jan 3;134(3):959–78. doi: 10.1007/s00122-020-03745-3 (PMC7925484; doi:10.1007/s00122-020-03745-3)
Supplement: Supplementary file 1 — Supplementary file2 (PDF 1206 kb) [file 122_2020_3745_MOESM1_ESM.pdf]

Genetic variability and genome-wide association analysis of flavor and texture in cooked beans (*Phaseolus vulgaris* L.)

Amber Bassett[1], Kelvin Kamfwa[2], Daniel Ambachew[34], Karen Cichy[15]*

[1] Department of Plant, Soil and Microbial Sciences, Michigan State University, East Lansing, MI, USA
[2] Department of Plant Science, University of Zambia, Lusaka, Zambia
[3] Southern Agricultural Research Institute, Hawassa, Ethiopia
[4] Department of Agricultural and Environmental Sciences, Tennessee State University, Nashville, Tennessee
[5] Sugarbeet and Bean Research Unit, USDA-ARS, East Lansing, MI, USA

*Corresponding Author: Phone: 517-353-0210; Email: karen.cichy@usda.gov

Supplementary Figures and Tables

Fig. S1 Manhattan and QQ plots for total flavor intensity, starchy intensity, bitter intensity, and cotyledon texture of the Andean Diversity Panel with mapping conducted using MLM with BLUPs from all locations combined. The gray dashed line is the $\alpha = 0.05$ Bonferroni correction based on the effective number of markers determined using the SimpleM algorithm.

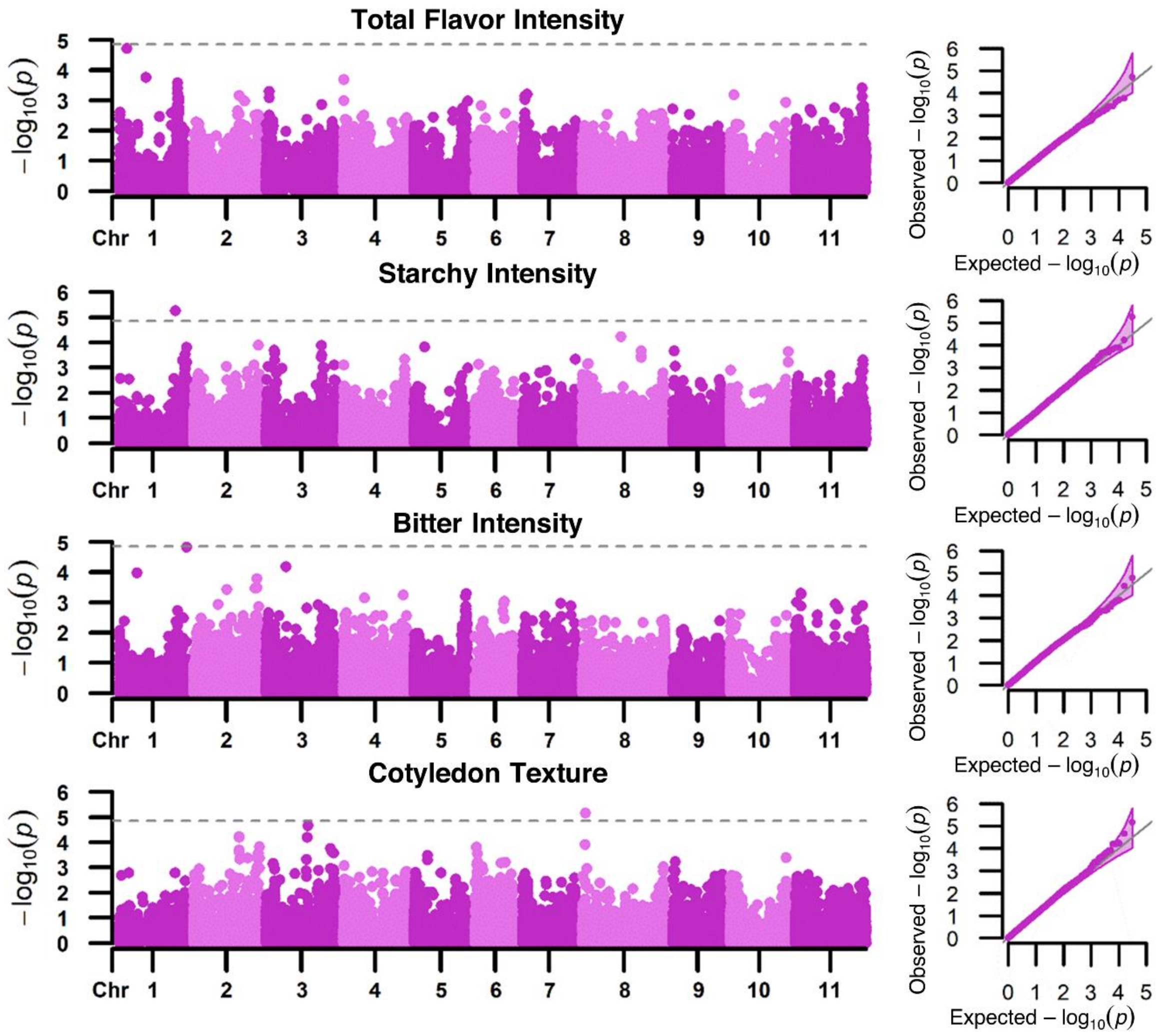

Fig. S2 Manhattan and QQ plots for total flavor intensity of the Andean Diversity Panel with mapping conducted using BLINK with BLUPs for Hawassa, Ethiopia (H); Kabwe, Zambia (K); and Lusaka, Zambia (L). The gray dashed line is the α = 0.05 FDR.

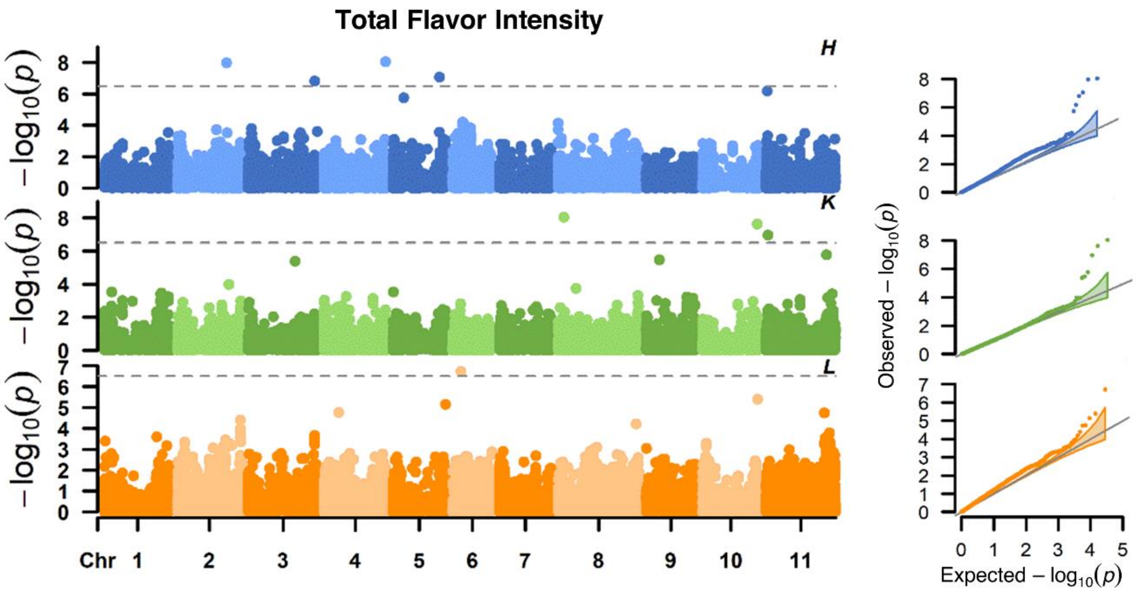

Fig. S3 Manhattan and QQ plots for beany intensity of the Andean Diversity Panel with mapping conducted using BLINK with BLUPs for Hawassa, Ethiopia (H); Kabwe, Zambia (K); and Lusaka, Zambia (L). The gray dashed line is the α = 0.05 FDR.

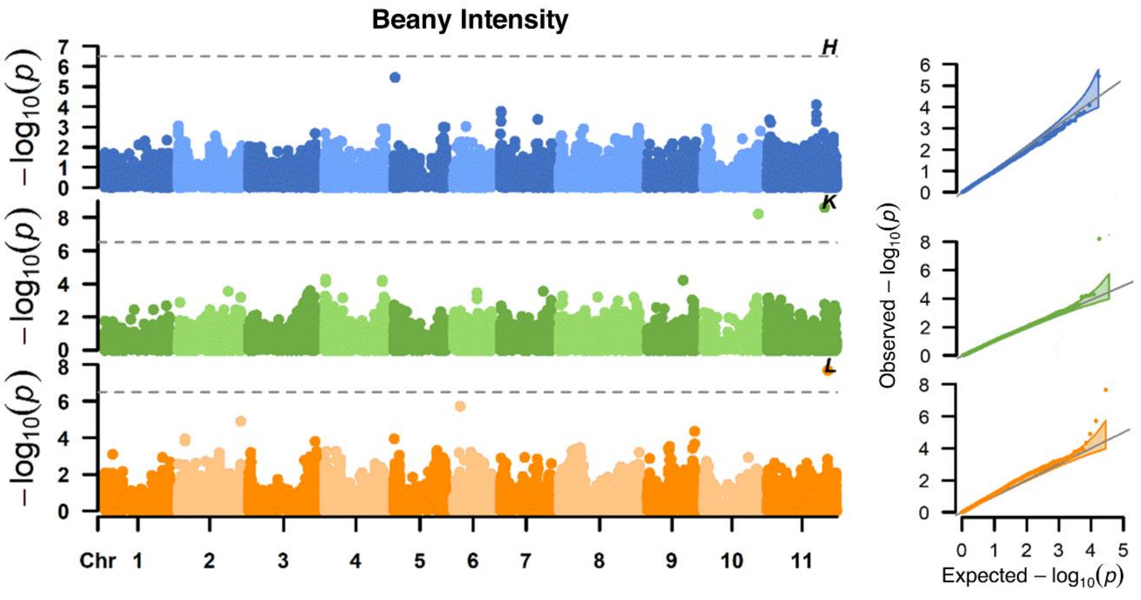

Fig. S4 Manhattan and QQ plots for earthy intensity of the Andean Diversity Panel with mapping conducted using BLINK with BLUPs for Hawassa, Ethiopia (H); Kabwe, Zambia (K); and Lusaka, Zambia (L). The gray dashed line is the α = 0.05 FDR.

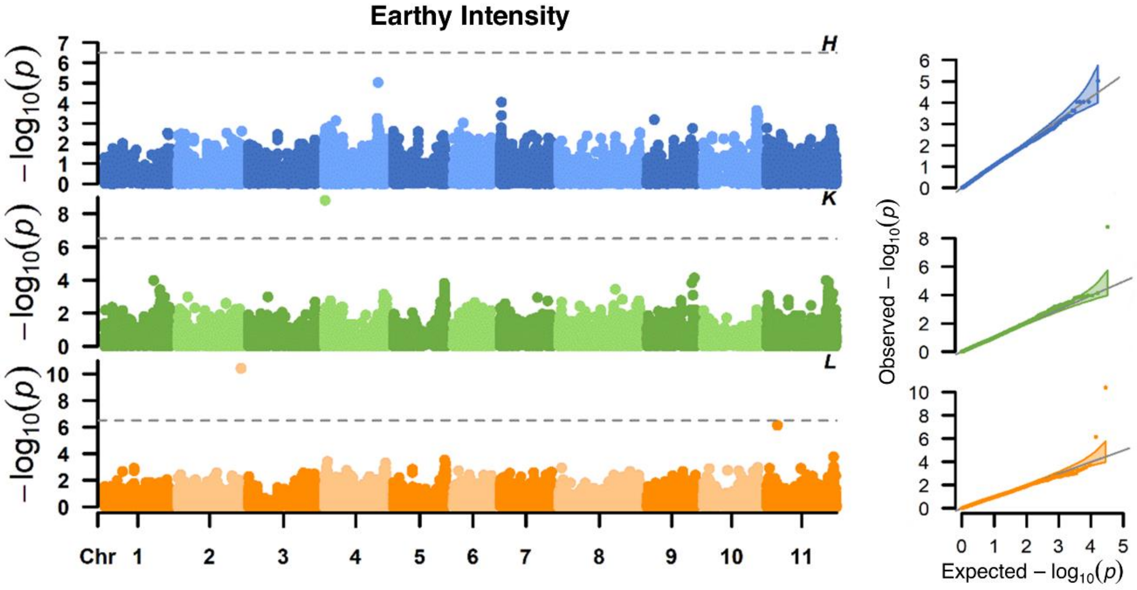

Fig. S5 Manhattan and QQ plots for seed-coat perception of the Andean Diversity Panel with mapping conducted using BLINK with BLUPs for Hawassa, Ethiopia (H); Kabwe, Zambia (K); and Lusaka, Zambia (L). The gray dashed line is the α = 0.05 FDR.

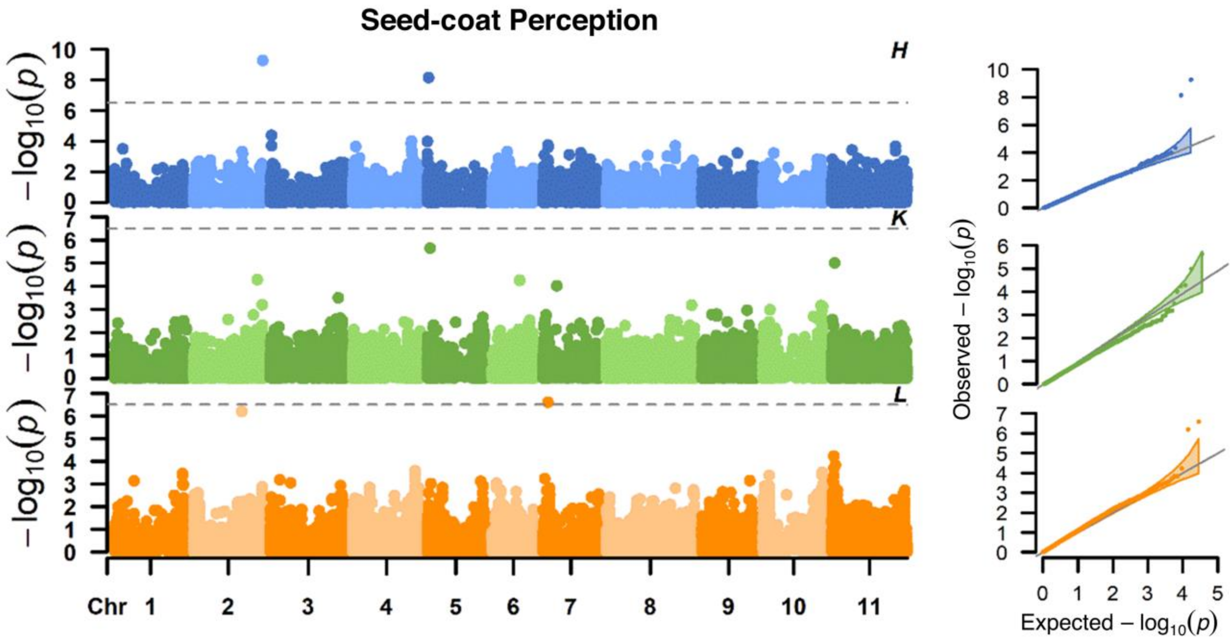

Fig. S6 Manhattan and QQ plots for raw seed weight, soak water uptake, cooking time, and total water uptake of the Andean Diversity Panel with mapping conducted using BLINK with BLUPs from all locations combined. The gray dashed line is the α = 0.05 FDR.

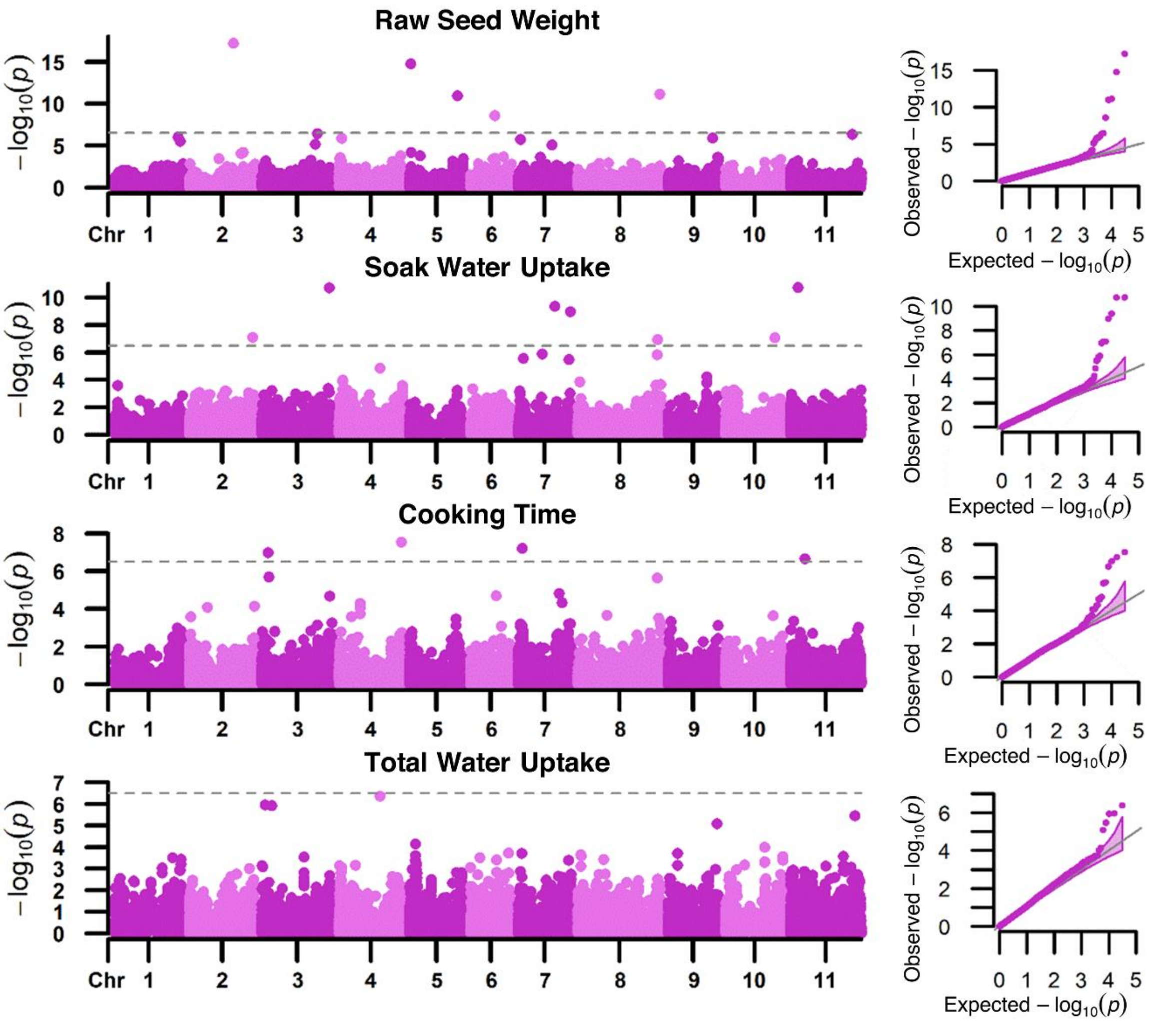

Fig. S7 Manhattan and QQ plots for raw seed weight, soak water uptake, and cooking time of the Andean Diversity Panel with mapping conducted using MLM with BLUPs from all locations combined. The gray dashed line is the α = 0.05 Bonferroni correction based on the effective number of markers determined using the SimpleM algorithm.

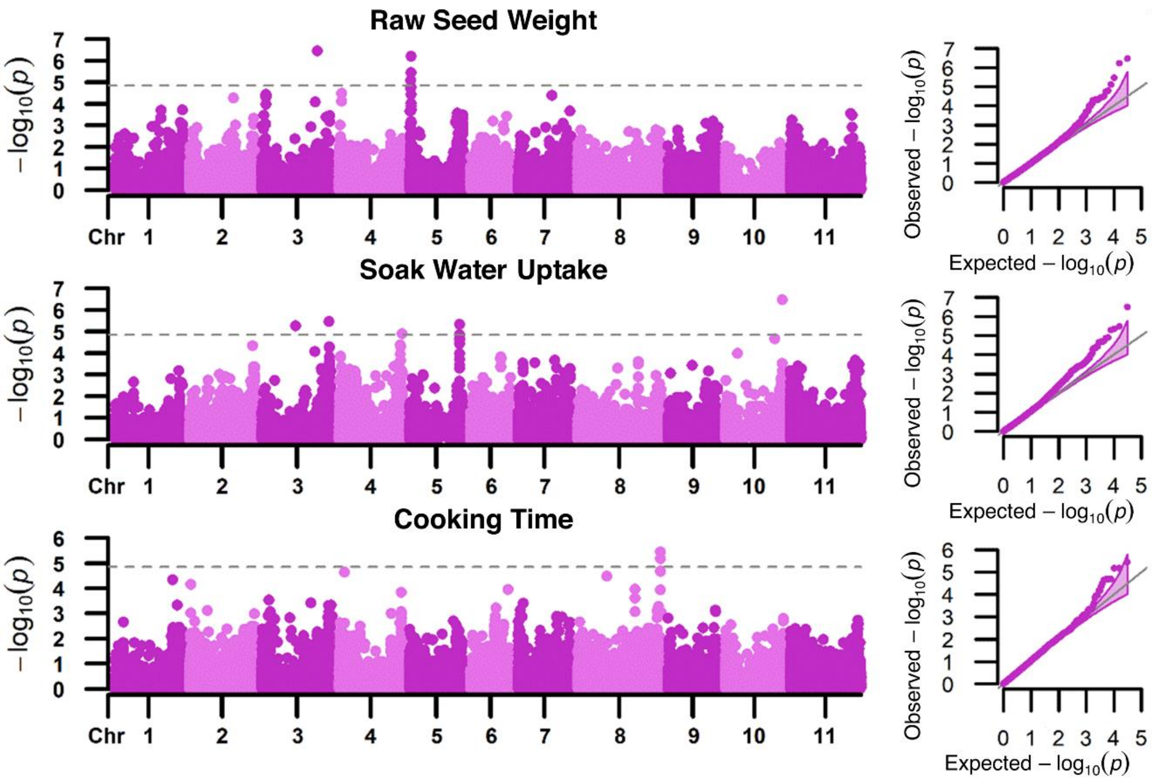

Fig. S8 Phenotypic effects of carrying the indicated number of significant markers conferring a positive effect for raw seed weight, soak water uptake, and total water uptake and a negative effect for cooking time. Phenotypic values represent all locations combined as averages from Hawassa, Ethiopia; Kabwe, Zambia; and Lusaka, Zambia. N is the number of individuals in each boxplot.

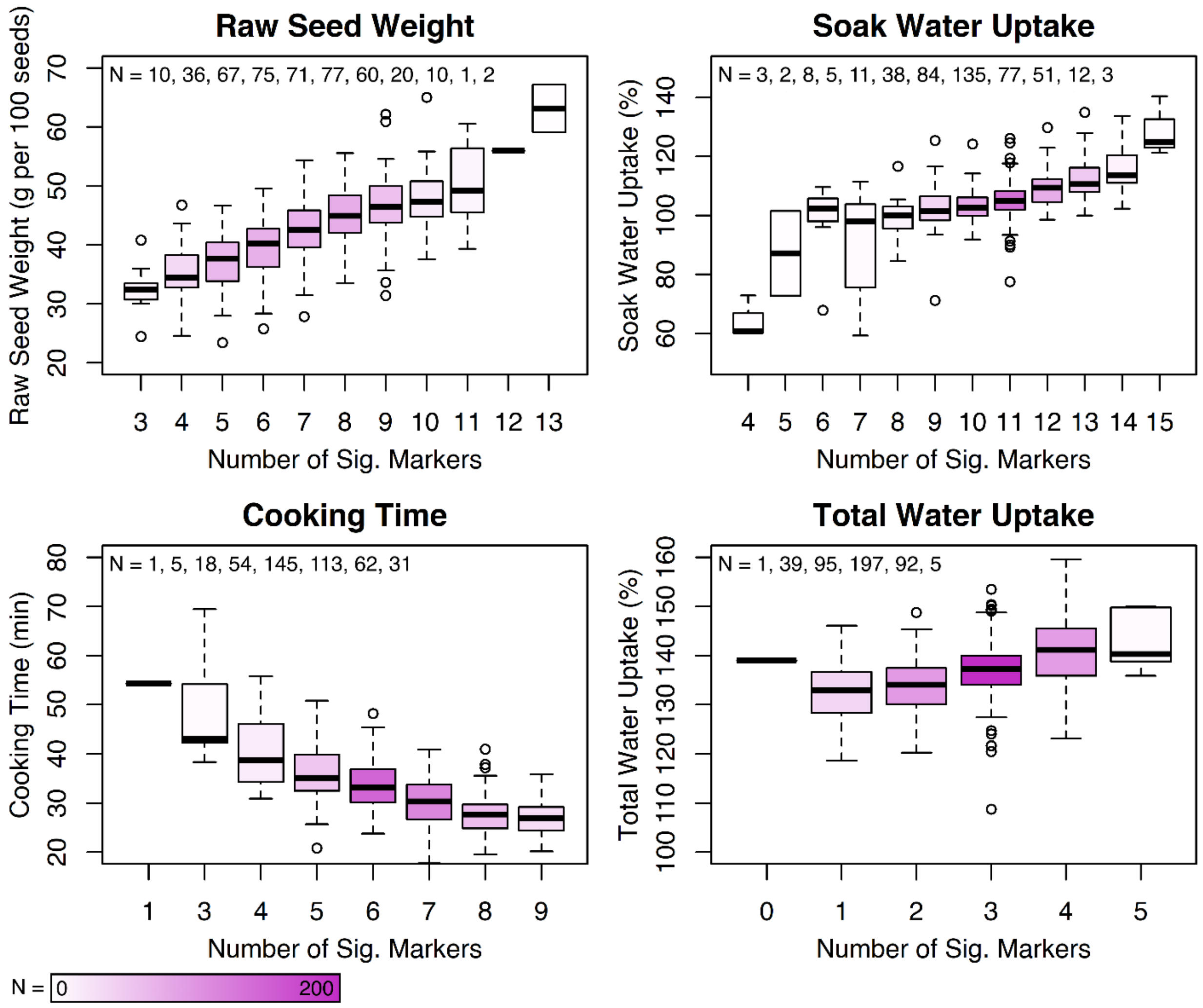

Table S1 Genotype information: Included in a separate file.

Table S2 5-point sensory attribute intensity scales.

Trait	Scale Description
Total Flavor Intensity	1-5, bland to strongly flavored
Beany Intensity	1-5, no/very little beany flavor to very strong beany flavor
Vegetative Intensity	1-5, no vegetative flavor to very strong vegetative flavor
Earthy Intensity	1-5, no earthy flavor to very strong earthy flavor
Starchy Intensity	1-5, no starchy taste to very strong starchy taste
Sweet Intensity	1-5, no sweet taste to very strong sweet taste
Bitter Intensity	1-5, no bitter taste to very strong bitter taste
Seed-coat Perception	1-5, imperceptible seed coat to very tough/lingering seed coat
Cotyledon Texture	1-5: mushy to very gritty/firm

Table S3 *P*-values[a] for the random effects from the sensory attribute intensity ANOVAs at the genotype level.

Trait	Rep	Panelist(Loc)	Session(Loc)
Total Flavor Intensity	NS	<0.0001	<0.0001
Beany Intensity	NS	<0.0001	<0.0001
Vegetative Intensity	NS	<0.0001	<0.0001
Earthy Intensity	NS	<0.0001	0.0004
Starchy Intensity	NS	<0.0001	<0.0001
Sweet Intensity	NS	<0.0001	<0.0001
Bitter Intensity	NS	<0.0001	0.0002
Seed-coat Perception	<0.0001	<0.0001	<0.0001
Cotyledon Texture	NS	<0.0001	<0.0001

[a] NS indicates non-significant *p*-values at $\alpha = 0.05$

Table S4 Least squares estimates of sensory attribute intensities across the 3 locations for the genotypes exhibiting extreme sensory attribute intensities[a].

ADP ID	Total Flavor	Beany	Vegetative	Earthy	Starchy	Sweet	Bitter	Seed-coat Perception	Cotyledon Texture
ADP0106	2.64	2.96	2.00	1.65	3.10	1.25	1.74	2.80	2.86
ADP0681	3.98	3.42	2.44	2.18	3.2	2.19	1.28	3.82	2.32
ADP0654	3.75	3.61	1.67	2.22	2.93	1.71	2.06	3.02	2.56
ADP0530	3.74	3.04	3.39	2.83	3.48	2.01	1.45	2.68	2.86
ADP0057	2.51	2.74	1.97	2.99	3.49	1.85	1.68	2.53	2.44
ADP0206	3.03	2.00	2.03	2.59	4.38	2.19	1.08	2.67	3.19
ADP0190	3.60	2.59	1.20	1.82	3.63	3.46	1.03	2.34	2.82
ADP0517	3.64	2.56	2.71	2.01	2.1	1.23	3.18	3.3	2.54
ADP0005	2.43	2.90	1.69	1.54	2.79	1.37	1.13	1.64	2.64
ADP0450	2.73	2.44	2.38	1.97	2.94	1.48	1.28	4.43	3.07
ADP0791	2.16	2.17	1.98	1.83	3.35	1.40	1.28	2.53	2.00
ADP0044	2.78	2.04	1.63	1.72	2.83	1.50	2.64	2.78	3.17

[a] Extreme attributes exhibited by each genotype are indicated with boxes

Table S5 *P*-values[a] for the fixed and random effects from the sensory attribute intensity ANOVAs at the seed type level.

Trait	Seed Type	Loc	Seed Type x Loc	Rep	Reviewer(Loc)	Session(Loc)
Total Flavor Intensity	<.0001	0.0001	<0.0001	0.0008	<0.0001	<0.0001
Beany Intensity	<.0001	NS	0.0002	0.0408	<0.0001	<0.0001
Vegetative Intensity	<.0001	NS	0.0006	NS	<0.0001	<0.0001
Earthy Intensity	<.0001	NS	0.0009	NS	<0.0001	<0.0001
Starchy Intensity	<.0001	NS	NS	NS	<0.0001	<0.0001
Sweet Intensity	<.0001	NS	<0.0001	0.0314	<0.0001	<0.0001
Bitter Intensity	<.0001	NS	0.0032	NS	<0.0001	<0.0001
Seed-coat Perception	<.0001	NS	0.0011	<0.0001	<0.0001	<0.0001
Cotyledon Texture	<.0001	0.0009	<0.0001	NS	<0.0001	<0.0001

[a] NS indicates non-significant *p*-values at $\alpha = 0.05$

Table S6 GWAS significant markers associated with sensory attribute intensities determined via BLINK with marker, chromosome (Chr), position, *P*-value, minor allele frequency (MAF), major and minor alleles (Maj/Min), significance (Sig), and location indicated.

Trait	Marker	Chr	Position[a]	*P*-value	MAF	Maj/Min[b]	Sig[c]	Loc[d]
Total Flavor Intensity								
	S02_37932341	2	37932341	1.04E-08	0.25	G/**A**	***	H
	S03_36213088	3	36213088	4.32E-06	0.18	T/**A**	**	K
	S03_51252684	3	51252684	1.57E-07	0.45	G/**A**	***	H
	S04_47465212	4	47465212	8.95E-09	0.12	**T**/C	***	H
	S05_8530078	5	8530078	1.84E-06	0.19	**T**/A	***	H
	S05_35951411	5	35951411	8.83E-08	0.09	**G**/A	***	H
	S05_40598752	5	40598752	7.14E-06	0.10	G/**T**	*	L
	S06_6583452	6	6583452	1.92E-07	0.42	**T**/A	***	L
	S08_4550936	8	4550936	9.09E-09	0.06	**C**/T	***	K
	S09_10273671	9	10273671	3.39E-06	0.32	**G**/A	**	K
	S10_42515259	10	42515259	2.38E-08	0.06	**T**/A	***	K
	S10_42798266	10	42798266	4.04E-06	0.11	**A**/G	*	L
	S11_726776	11	726776	6.73E-07	0.25	G/**A**	***	H
	S11_1465049	11	1465049	1.12E-07	0.24	**C**/T	***	K
	S11_46750806	11	46750806	1.72E-06	0.25	G/**T**	**	K
Beany Intensity								
	S02_48688740	2	48688740	1.29E-05	0.07	A/**G**	*	L
	S06_5391064	6	5391064	1.93E-06	0.07	**T**/G	**	L
	S10_42528848	10	42528848	6.12E-09	0.12	**G**/A	***	K
	S10_44117615	10	44117615	4.69E-06	0.06	**C**/T	**	L
	S11_44125952	11	44125952	2.57E-09	0.07	C/**A**	***	K
	S11_46580267	11	46580267	2.21E-08	0.16	**A**/C	***	L
Earthy Intensity								
	S02_48899330	2	48899330	4.00E-11	0.12	A/**C**	***	L
	S04_448769	4	448769	1.56E-09	0.08	C/**T**	***	K
	S11_8151131	11	8151131	7.34E-07	0.09	A/**G**	**	L
Seed-coat Perception								
	S02_34387999	2	34387999	6.35E-07	0.06	G/**T**	***	L
	S02_49203869	2	49203869	5.40E-10	0.28	G/**A**	***	H
	S05_1034657	5	1034657	7.00E-09	0.37	**T**/A	***	H
	S05_2198768	5	2198768	2.29E-06	0.44	**C**/G	*	K
	S07_3664145	7	3664145	2.50E-07	0.49	G/**C**	***	L

[a] Position is based on the P. vulgaris v2.1 reference genome (DOE-JGI and USDA-NIFA, http://phytozome.jgi.doe.gov/)
[b] Alleles in bold confer a positive effect on the indicated trait

[c] Significance is indicated by asterisks, such that *, **, *** indicate significance at $\alpha = 0.1$, $\alpha = 0.05$, $\alpha = 0.01$ using the false discovery rate
[d] H is Hawassa, Ethiopia; K is Kabwe, Zambia; and L is Lusaka, Zambia

Table S7 GWAS significant markers associated with cooking time, soak water uptake, raw seed weight, and total water uptake, with chromosome (Chr), position, *P*-value, minor allele frequency (MAF), major and minor alleles (Maj/Min), significance (Sig), and method indicated.

Trait	Marker	Chr	Position[a]	*P*-value	MAF	Maj/Min[b]	Sig[c]	Method
Raw Seed Weight								
	S01_47840887	1	47840887	9.04E-07	0.14	**G**/A	***	BLINK
	S01_49584124	1	49584124	3.14E-06	0.47	C/**G**	***	BLINK
	S02_33254640	2	33254640	5.72E-18	0.06	**T**/A	***	BLINK
	S03_40318649	3	40318649	6.57E-06	0.30	**G**/A	**	BLINK
	S03_41895570	3	41895570	3.55E-07	0.31	A/**C**	***	BLINK, MLM
	S04_1769598	4	1769598	1.36E-06	0.12	G/**A**	***	BLINK
	S05_1069847	5	1069847	1.79E-15	0.49	**A**/G	***	BLINK
	S05_1138961	5	1138961	6.10E-07	0.36	C/**T**	**	MLM
	S05_36225413	5	36225413	1.05E-11	0.15	T/**C**	***	BLINK
	S06_18456447	6	18456447	2.59E-09	0.08	G/**A**	***	BLINK
	S07_1842933	7	1842933	1.91E-06	0.18	**C**/A	***	BLINK
	S07_25513414	7	25513414	8.08E-06	0.18	T/**C**	**	BLINK
	S08_61954787	8	61954787	7.27E-12	0.39	A/**G**	***	BLINK
	S09_33770475	9	33770475	1.27E-06	0.07	**A**/G	***	BLINK
	S11_46634045	11	46634045	4.36E-07	0.13	G/**A**	***	BLINK
Soak Water Uptake								
	S02_47837868	2	47837868	8.17E-08	0.25	**G**/A	***	BLINK
	S03_25546920	3	25546920	5.30E-06	0.05	**C**/T	*	MLM
	S03_50652595	3	50652595	3.36E-06	0.07	**A**/T	**	MLM
	S03_51140861	3	51140861	1.89E-11	0.07	**G**/A	***	BLINK
	S04_30764016	4	30764016	1.41E-05	0.24	C/**T**	**	BLINK
	S04_47654443	4	47654443	1.26E-05	0.08	**G**/A	*	MLM
	S05_37924556	5	37924556	4.53E-06	0.07	**C**/G	**	MLM
	S07_3919560	7	3919560	2.66E-06	0.06	**C**/G	***	BLINK
	S07_18212326	7	18212326	1.34E-06	0.09	**A**/G	***	BLINK
	S07_27774103	7	27774103	4.22E-10	0.19	C/**T**	***	BLINK
	S07_38497123	7	38497123	3.20E-06	0.38	**A**/G	***	BLINK
	S07_39390008	7	39390008	1.10E-09	0.42	A/**G**	***	BLINK
	S08_59981977	8	59981977	1.49E-06	0.05	**T**/A	***	BLINK
	S08_60478317	8	60478317	1.14E-07	0.34	C/**T**	***	BLINK
	S10_37637761	10	37637761	8.68E-08	0.12	**T**/C	***	BLINK,MLM
	S10_43391440	10	43391440	3.25E-07	0.06	**A**/G	**	MLM
	S11_5714496	11	5714496	1.83E-11	0.05	**C**/A	***	BLINK
Cooking Time								
	S03_4885990	3	4885990	1.03E-07	0.05	T/**G**	***	BLINK
	S03_5243893	3	5243893	2.04E-06	0.07	A/**G**	*	BLINK

S03_51292502	3	51292502	2.07E-05	0.06	A/**T**	*	BLINK
S04_3957256	4	3957256	2.24E-05	0.24	**C**/G	*	MLM
S04_47068842	4	47068842	2.93E-08	0.08	A/**G**	***	BLINK
S06_19636517	6	19636517	2.02E-05	0.08	**T**/G	*	BLINK
S07_3009718	7	3009718	6.05E-08	0.07	T/**C**	***	BLINK
S07_30919254	7	30919254	1.54E-05	0.35	**T**/C	*	BLINK
S08_60104796	8	60104796	2.31E-06	0.27	**C**/A	**	BLINK
S08_62659170	8	62659170	3.52E-06	0.16	A/**G**	**	MLM
S11_10805992	11	10805992	2.22E-07	0.10	**C**/T	***	BLINK
Total Water Uptake							
S03_2580077	3	2580077	1.11E-06	0.07	G/**A**	**	BLINK
S03_7619818	3	7619818	1.17E-06	0.27	**T**/C	**	BLINK
S04_30764016	4	30764016	4.25E-07	0.24	C/**T**	**	BLINK
S09_37046204	9	37046204	8.29E-06	0.08	**C**/T	*	BLINK
S11_48753729	11	48753729	3.44E-06	0.12	**T**/A	**	BLINK

[a] Position is based on the P. vulgaris v2.1 reference genome (DOE-JGI and USDA-NIFA, http://phytozome.jgi.doe.gov/)
[b] Alleles in bold confer a positive effect on the indicated trait
[c] Significance is indicated by asterisks, such that *, **, *** indicate significance at α = 0.1, α = 0.05, α = 0.01 using the false discovery rate for the BLINK method and a Bonferroni correction based on the effective number of markers determined using the SimpleM algorithm for the MLM method
